# Guidelines for the management of extravasation

**DOI:** 10.3352/jeehp.2020.17.21

**Published:** 2020-08-10

**Authors:** Jung Tae Kim, Jeong Yun Park, Hyun Jung Lee, Young Ju Cheon

**Affiliations:** 1 Department of Pharmacy, Kyung Hee University Hospital at Gangdong, Seoul, Korea; 2 Department of Clinical Nursing, University of Ulsan, Seoul, Korea; Hallym University, Korea

**Keywords:** Extravasation, Antidotes, Intravenous injections, Patient care, Risk factors

## Abstract

The purpose of these practice guidelines is to offer and share strategies for preventing extravasation and measures for handling drugs known to cause tissue necrosis, which may occur even with the most skilled experts at intravenous (IV) injection. Herein, general knowledge about extravasation is first described, including its definition, incidence, risk factors, diagnosis, differential diagnosis, and extravasation injuries. Management of extravasation includes nursing intervention and thermal application. At the first sign of extravasation, nursing intervention with following steps is recommended: stop administration of IV fluids immediately, disconnect the IV tube from the cannula, aspirate any remaining drug from the cannula, administer drug-specific antidote, and notify the physician. Local thermal treatments are used to decrease the site reaction and absorption of the infiltrate. Local cooling (ice packs) aids in vasoconstriction, theoretically limiting the drug dispersion. Although clear benefit has not been demonstrated with thermal applications, it remains a standard supportive care. The recommended application schedule for both warm and cold applications is 15 to 20 minutes, every 4 hours, for 24 to 48 hours. For prevention of extravasation, health professionals should be familiar with the extravasation management standard guidelines. They should regularly check the extravasation kit, assess patients’ sensory changes, tingling or burning, and always pay attention to patients’ words. The medical team’s continuous education on extravasation is essential. With the practical use of these guidelines, it is expected to reduce the occurrence rate of extravasation and contribute to patient care improvement.

## Introduction

Extravasation refers to the leakage of injected drugs from blood vessels causing damage to the surrounding tissues. Common symptoms and signs of extravasation include pain, stinging or burning sensations, and edema around the intravenous (IV) injection site. In severe cases, extravasation may cause tissue dysfunction or physical defects, resulting in a delay of attempted treatment, patients’ distrust, and numerous other issues. To prevent extravasation, a clinical specialist should perform the venipuncture or injection, who with relevant skills and management ability understands the properties of the injected drug. The primary purpose of these guidelines is to minimize the side-effects of IV injection, by suggesting proper and prompt emergency measures for extravasation and the appropriate treatments corresponding to the properties of the injected drug. The second purpose is to raise the medical team’s awareness of extravasation in order to prevent extravasation with careful injection, recover patient trust, and increase patient satisfaction. These guidelines consist of following topics: basic knowledge about extravasation, extravasation management, and extravasation prevention. Antidotes, special drug management, drugs with high osmolarity, and drugs with pH are provided as supplement files (Supplements 1–4). These contents are derived from authors’ experiences and the references [1-21]. It is anticipated that these guidelines would help health professionals to prevent extravasation during IV and central vein injection and to promote patient safety should extravasation occur in any case.

## Extravasation

### Definition

Extravasation is the leakage of an injected drug out of the blood vessels, damaging the surrounding tissues. In terms of cancer therapy, extravasation refers to the inadvertent infiltration of chemotherapeutic drugs in the tissues surrounding the IV site.

Extravasated drugs are classified according to their potential for causing damage as ‘vesicant,’ ‘irritant,’ and ‘nonvesicant.’ Vesicant drugs are also classified into 2 groups: DNA binding and non-DNA binding.

### Incidence

The frequency of extravasation in adults is reported to be between 0.1% and 6%. Some data suggest that the incidence is decreasing probably due to improvements in the infusion procedure, early recognition of the drug leakage, and training in management techniques.

### Risk factors

Risk factors can be classified under patient-related, procedure-related, and product or product-related factors.

#### Patient-related factors

- Small and fragile veins in infants, children, or elderly patients

- Vessels that may burst easily

- Cancer patients with hardened and thickened vessels due to frequent venipuncture

- Patients with vessels that move easily during venipuncture attempts

- Patients with excised lymph nodes, limb amputation, or closed vena cava

- Obesity in which peripheral venous access is more difficult

- Patients who move around a lot

#### Procedure-related factors

- Untrained or inexperienced staff

- Multiple attempts at cannulation

- High flow pressure

#### Product or product-related factors

- Inadequate choice of equipment (peripheral catheter choice, size, or steel needle)

- Inadequate dressings

- Poor cannula fixation

### Diagnosis

Patients must be informed to report any changes in sensation, signs, or symptoms during the IV administration of any chemotherapeutic drug and to alert the healthcare professionals to early signs of extravasation. Particular information must be given when a vesicant drug is administered. Extravasation must be suspected if any of the following specific signs or symptoms are presented (Table 1).

#### In the case of peripheral IV catheter

- Possibly no initial symptoms of extravasation

- Redness, pruritus, and edema around the injection site

- Fluid injection rate slows down or stops

- Blood backflow does not work well or there is leakage of medication around the needle

- A complaint of discomfort or pain and occasional expression of searing pain or numbness

- Initial physical symptoms usually appear immediately but also might appear several days or weeks later.

#### In the case of central venous catheter

- Often causes stinging pain

- Edema around the port insertion or in the chest, or medication leakage around the catheter insertion

- Redness in the chest, collarbone, or neck where a central venous catheter is inserted

- No blood backflow

- Symptoms may appear early or late.

### Differential diagnosis

#### Flare reaction

Spots or solid lines with blisters can be suddenly felt along the vessels injected with drugs. Pain, edema, and ulcer do not appear, and symptoms disappear within 30 to 90 minutes.

#### Vessel irritation

Pain, tightening, and skin discoloration tend to worsen. Blood backflow works well, and edema or ulcer do not occur. Pain or tightening occurs along the vein, and it is caused mainly by drugs such as vinorelbine and dacarbazine. Hot fomentations can be applied to the dilated veins to mitigate the symptoms.

#### Venous shock

Occurs due to contraction of the vessel wall and usually happens as soon as the fluid injection begins. For the most part, blood does not backflow. Discoloration and edema do not occur. Venous shock can occur when injecting very cold medication or when medication is injected at a rapid pace. Hot fomentations can dilate the veins and mitigate the symptoms.

### Extravasation injuries

While the injury is usually minor and resolves spontaneously, some cases result in serious complications, including full-thickness skin loss and muscle and tendon necrosis requiring reconstructive surgery or even amputation, leading to longer hospital stays, increased morbidity, and increased costs.

#### Pain

Narcotic analgesics may be required to reduce severe pain around widespread extensive necrosis.

#### Physical defects

Patients may be unable to work for some time; quality of life must be compensated for if a patient’s occupation requires full physical mobility, and exposure of the disfigurement in public can cause a psychological impact.

#### Medical expense

Depending on the situation, patients will bear the cost of hospitalization and medical expenses for cosmetic surgeries, and secondary medical problems might occur if the condition worsens.

#### Disease control

Treatment suspension wastes time and other problems can occur due to delayed treatment. If bone marrow function decreases, anticancer treatments may be delayed due to infection caused by leukopenia.

#### Time

The patient’s normal activities, such as at home, work, school, etc., may be disrupted until the patient is fully recovered.

#### Psychological impact on the nurse and the patient

Therapists will always feel nervous during the medical team-patient communication because of guilt. Communication and trust between patients and nurses can be interfered due to extravasation.

## Management of extravasation

### Nursing interventions

**Fig. 1.**
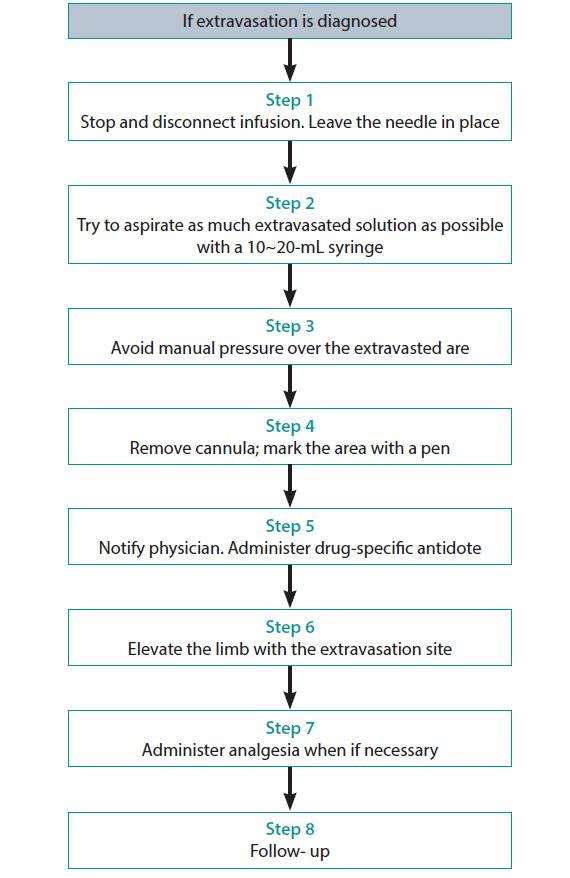
Steps to be taken of the extravasation.

At the first sign of extravasation, the following steps are recommended: (1) stop administration of IV fluids immediately, (2) disconnect the IV tube from the cannula, (3) aspirate any residual drug from the cannula, (4) administer a drug-specific antidote, and (5) notify the physician (Fig. 1).

Elevation of the limb may aid in reabsorption of the infiltrate or extravasated vesicant by decreasing capillary hydrostatic pressure. Apply sterile dressing over the area of extravasation, regularly assess the extravasation site during every shift, and take medical photographs and consult the department of cosmetic surgery if necessary.

### Thermal application

Local thermal treatments are used to decrease the site reaction and absorption of the infiltrate. Local cooling (ice packs) aids in vasoconstriction, theoretically limiting the drug dispersion. Cold application is recommended for extravasation of DNA-binding vesicants except for mechlorethamine (nitrogen mustard), contrast media, and hyperosmolar fluids. The use of local warming therapy (dry heat) is based on the theory that it enhances vasodilation, thus enhancing the dispersion of the vesicant agent and decreasing drug accumulation in the local tissue. The use of local warming is recommended for the extravasation of non–DNA-binding vesicants. Although clear benefit has not been demonstrated with thermal applications, it remains a standard supportive care, and the recommended application schedule for both warm and cold applications is 15 to 20 minutes, every 4 hours, for 24 to 48 hours.

#### Local cooling

- It causes contraction of blood vessels, minimizing the spread of drugs to other tissues and reducing topical infections and pain.

- Directions: apply cold fomentations for 15 to 20 minutes four to 6 times per day (for 1 day or more).

#### Local warming

- It dilates the blood vessels around the extravasation site, increases dispersion and absorption of the medicinal fluid by increasing the blood flow, and helps to quickly purge medicinal fluid that has leaked from the extravasation site.

- Directions: apply hot fomentations for 20 to 30 minutes four to 6 times per day (for 1 day or more).

### Documentation

Because errors associated with IV administration can result in fatal or life-threatening outcomes, administration of IV fluids and medications can be a high-risk, with adverse outcomes potentially leading to malpractice claims.

An incident of extravasation must be correctly documented and reported. Documentation procedure may differ between treatment centers (documentation form); however, certain items are mandatory for patient safety and legal purposes: (1) patient name and number, (2) date and time of the extravasation, (3) name of the drug extravasated and the diluent used (if applicable), (4) signs and symptoms (also reported by the patient), (5) description of the IV access, (6) extravasation area (and the approximate amount of the drug extravasated), and (7) management steps with time and date.

Photographic documentation can be helpful for follow-up procedures. The patient must be informed of the scope of the problem (Supplement 5).

## Prevention of extravasation

### General guidelines

Most extravasations can be prevented with the systematic implementation of careful, standardized, and evidence-based administration techniques. The staff involved in the infusion and management of cytotoxic drugs must be trained to implement several preventive protocols for the minimization of the risk of extravasation. It is important to remember that the degree of damage is dependent on the type of the drug, the drug concentration, the localization of the extravasation, and the length of time for which the drug develops its potential for damage.

- Be familiar with the extravasation management standard guidelines and prepare the extravasation kit.

- Regularly check the extravasation kit and refill any used medications. Extravasation kit includes the following: 25G needle, 10-cc syringe, and 1-mL syringe; disinfection swabs, sterile gauze, and adhesive bandage; saline solution (1 ampule); sterile distilled water (1 ampule); dimethyl sulfoxide 99% solution; hyaluronidase 1,500 U/mL (refrigerated); hydrocortisone cream 1%; sodium thiosulfate 25% solution; and warm pack and an ice pack (frozen).

- Assess patient’s sensory changes, tingling or burning, and always pay attention to the words of patients.

### Preventive strategies: peripheral venous access device extravasation

- Do not insert the cannula in the joints because it is difficult to secure, and neural damage and tendon injury can be caused if extravasation occurs due to vesicant drugs.

- Do not insert the cannula in the antecubital fossa area, where it is extremely difficult to detect extravasation.

- Veins on the back of the hand can be used, and in some cases, observation is easier. But it must be done carefully because this area can suffer a more severe injury due to extravasation.

- For observation, do not cover the cannula area with opaque gauze.

- Secure the cannula during the administration of the drug.

- Even if there is an existing IV route, secure a new route when administering vesicant drugs.

- If in doubt, re-insert the cannula and administer the drug.

- Watch for edema, inflammation, and pain around the cannula during administration.

- Check for blood backflow before/during administration, and always rinse the catheter with a saline solution in between administrations.

- Dilute stimulant drugs as much as possible and inject them at a proper rate.

- Once the needle is removed, apply pressure to the puncture site for about five minutes and elevate the limb.

### Preventive strategies: central venous access device extravasation

- Check for blood backflow before injection to ensure that the catheter is positioned in the vein.

- Check if there is any local discomfort or swelling by running a saline solution through the catheter, and then inject the drug.

- After the injection, make sure to run a saline solution through the catheter.

## Conclusion

Extravasation is a serious complication during patient care. Although drugs can be administered by methods (e.g., micro-patch, micro-injection) other than IV injection, extravasation cannot be totally avoided because there are drugs that can only be administered through IV or central vein injection. The guidelines described herein are based on the authors’ best practice for the management and prevention of the extravasation. However, no guidelines can be perfect, and they need to be regularly updated. It will be our pleasure if these guidelines are used in the training of health professionals to promote patients’ safety.

## Figures and Tables

**Table 1.** Extravasation assessment tool

	Grade				
	0	1	2	3	4
Color	Normal	Pink	Red	Blanched	Blackened
Integrity	Unbroken	Blistered	Superficial skin loss	Tissue loss exposing subcutaneous tissue	Tissue loss exposing muscle/bone with a deep crater or necrosis
Skin temperature	Normal	Warm	Hot		
Edema	Absent	Non-pitting	Pitting		
Mobility	Full	Slightly limited	Very limited	Immobile	
Pain	Rate on a scale of 0–10				
Fever	Normal	Elevated (highest value during 24 hours)			

## Supplementary materials

Data files are available from Harvard Dataverse: https://doi.org/10.7910/DVN/NUWZML

Supplement 1. Antidotes for the care of the extravasation.

Supplement 2. Special drug management for care of the extravasation.

Supplement 3. Drugs with high osmolarity.

Supplement 4. Drugs with pH.

Supplement 5. Photographic documentation of extravasation case.

Supplement 6. The audio recording of the abstract.
