# Supplementary material for: Guidelines for the management of extravasation
Source: J Educ Eval Health Prof. 2020 Aug 10;17:21. doi: 10.3352/jeehp.2020.17.21 (PMC7431942; doi:10.3352/jeehp.2020.17.21)
Supplement: Supplementary file 1 — Supplement 1. Antidotes for the care of the extravasation. [file jeehp-17-21-suppl1.docx]

**Supplement 1. Antidotes**

Extravasation can be caused during the administration process, depending on the injection site, venipuncture techniques, or skills. Extravasation can also occur in elderly patients, patients with cardiovascular disease, or patients with phlebitis. In addition, extravasation can occur when the properties of drugs are necrosis-inducing, when the difference of pH levels and osmolarities is large, as in 5-fluorouracil, whose pH-value is close to 9 if not diluted, or when a large amount of highly concentrated medication is administered intravenously.

| Drug-associated risk factors  Necrotizing capacity of the agent  Amount of extravasated agent (i.e. concentration in the solution and volume)  Duration of exposure to extravasation  pH value  Osmolarity  Excipients such as benzyl alcohol (local anesthetic effect) |
| --- |

Therefore, extravasation-inducing drugs are classified mainly into cytotoxic drugs and non-cytotoxic drugs. Depending on tissue damage potency, cytotoxic drugs are categorized into vesicants, exfloriants, irritants, and non-vesicants (or inflammitants and neutrals).

1. Cytotoxic Agents

***Vesicants***

The term “vesicants” refers to substances that are capable of causing pain, inflammation and blistering of the local skin, underlying structures, leading to tissue death and necrosis. And among them, anthracyclines, vinca alkaloids, and mitomycin C are representative. Depending on the action mechanism, vesicants are divided into DNA-binding and non-DNA-binding. DNA-binding anthracyclines are combined with nucleic acid to inhibit topoisomerase II to cut out the DNA strand, and generate free radicals to inhibit protein synthesis with RNA to cause cell necrosis. Non-DNA-binding vinca alkaloids (vincristine, vinblastine, and vinorelbine) show cytotoxic effects by inhibiting the generation of microtubule.2) In the case of DNA binding agents, vascular leakage can cause combination with the nucleic acid within the cells, resulting in spreading of toxicity to surrounding tissue, and the degree of damage can be much more severe. Therefore, the treatment processes for extravasation are also different between DNA-binding and non-DNA binding.

Anthracyclines (DNA-binding) can be administered with DMSO or dexrazoxane depending on the amount of extravasation in order to minimize the spreading of medicinal fluid and prevent the progression of necrosis. Cold compression must be used, and hydrocortisone 1% cream must be applied in order to inhibit inflammation.

DNA-binding Vesicant: **Localize and Neutralize**.

Apply topical DMSO immediately when extravasation occurs. Do not use if blistering present.

Allow the DMSO to dry, and then cover with a non-occlusive gauze dressing, this should be applied within 10-25 minutes.

Apply a cold pack for 30 minutes. Repeat every 4 hours for 24 hours to help localize the infusate.

Apply hydrocortisone 1% cream after 3 hours from the initial application of DMSO.

Elevate the limb

Non-DNA-binding Vesicant: **Disperse and Dilute**.

Hyaluronidase should begin within 1 hour of extravasation for best results.

Give several subcutaneously (or intradermal) injections of 150 IU of hyaluronidase diluted in 1 mL sterile water as 5 separate 0.2 mL injection around the periphery extravasated area.

Apply hydrocortisone 1% cream every 6 hours for as long as erythema persists.

Elevate the limb with the extravasation site.

Apply warm pack to the affected area for 30 minutes 4 times daily for 1 to 2 days.

***Exfoliants and Irritants***

“Exfoliants” means drugs which are capable of causing inflammation and shedding of the skin but less likely to cause tissue death. And “irritants” refers to drugs which are capable of causing inflammation, irritation or pain at site of extravasation but rarely cause tissue breakdown. In most cases, the related symptoms disappear within days or weeks. The symptoms rarely progress in the long term. Of anticancer drugs, platinum compounds and topoisomerase I inhibitors belong here.

**Exfoliants & Irritants: Localize**

Apply cold pack for 30 minutes every 4 hours for 24 hours

Apply hydrocortisone 1% cream every 6 hours for 7 days or as long as in the case of erythema persists.

***Non-Vesicants***

Drugs that belong to non-vesicants do not cause ulcers. These drugs are capable of causing mild to moderate inflammation and flare in local tissues. In fact, even in the case of extravascular leakage, it rarely progresses to an acute reaction or necrosis.

**Non-vesicants: Symptomatic Relief**

Elevate the limb

Consider applying a cold pack if local symptoms occur

Apply hydrocortisone 1% cream every 6 hours each day if erythema is present

***Classification of Cytotoxic Drugs and Compression***

Table 1. The classification of cytotoxic agents according to the potential to cause tissue damage.

| **Vesicants** | **Exfoliants** | **Irritants** | **Inflammitants** | **Neutrals** |
| --- | --- | --- | --- | --- |
| Amsacrine  Carmustine  Dacarbazine  Dactinomycin  Daunorubicin  Doxorubicin  Epirubicin  Idarubicin  Mechlorethamine  Mitomycin C  Paclitaxel  Streptozocin  Trabectedin  Vinblastine  Vincristine  Vindesine  Vinorelbine | Cisplatin*  Docetaxel  Liposoma  Doxorubicin  Liposoma  *Daunorubicin*  Mitoxantrone  Oxaliplatin  *Topotecan* | Bendamustine  Carboplatin*  Etoposide  Irinotecan  Temsirolimus  Teniposide | Fluorouracil  Methotrexate  Raltitrexed | Arsenic trioxide  Asparaginase  Bevacizumab  Bleomycin  Bortezomib  Cetuximab  Cladribine  Cyclophosphamide  Cytarabine  Eribulin  Fludarabine  Gemcitabine  Interferons  Interleukin-2  Melphalan  Pemetrexed  Pentostatin  Rituximab  Thiotepa  Trastuzumab |

**RED: Warm pack, BLUE: Cold pack, BLACK: none, (*see part III. Special management)**

2.  Non-Cytotoxic Agents

Non-cytotoxic drugs (other than anticancer drugs) are also vesicatory and can cause tissue necrosis. Non-cytotoxic drugs include hyperosmotic fluid that causes compartment syndrome, highly-concentrated electrolyte solution that causes ischemia by extending the depolarization of the muscles, sodium bicarbonate that changes pH within the cells, and vasopressors.

***Classification of Non-Cytotoxic Drugs and Compression***

Table 2. The common non-cytotoxic drugs causing extravasation.

| Vasopressors | Hyper-/Hypo-osmolar agents | Acidic/Alkaline agents |
| --- | --- | --- |
| Dopamine  Dobutamine  **Epinephrine**  Methylene blue  Norepinephrine  **Phenylephrine**  Vasopressin | Total Parenteral Nutrition  Calcium chloride 10% (2040 mOsm/L)  Calcium gluconate (669 mOsm/L)  Radiographic contrast media    *Others*  Ampicillin (566 mOsm/L)  Arginine (950 mOsm/L)  Dextrose 10–50% (504–2,520 mOsm/L)  Hypertonic sodium chloride  Mannitol 10% & 20% (11369 mOsm/L)  Nafcillin (363 mOsm/L)  Potassium (60 mEq/L=763 mOsm/L)  Potassium phosphate  Prostaglandins  Sodium bicarbonate 8.4% (2,000 mOsm/L)    *Containing propylene glycol*  Etomidate, lorazepam, diazepam  nitroglycerin, digoxin, phenytoin,  phenobarbital | Acyclovir (pH 11)  Aminophylline (pH 8.8–10)  Amiodarone (pH 3.5–4.5)  **Amphotericin B (pH 5–6)**  Cefotaxime  Co-trimoxazole  Diazepam  Digoxin  Doxycycline (pH 1.8–3.3)  Erythromycin  **Etomidate (pH 3–4)**  Foscarnet sodium  Ganciclovir  Pentamidine (pH 6.5)  Phenytoin (pH 10–12)  Promethazine (pH 4.0–5.5)  Sodium thiopental (pH 10–11)  Vancomycin (pH 4.0) |

**RED: Warm pack, BLUE: Cold pack**

***Vasopressors***

Vasopressor infiltration leads to high intradermal concentrations of the vasopressor into the local tissue. As a result, direct α-adrenergic-mediated vasospasm of the smaller veins and the vasa vasorum ensues, leading to inadequate distal blood flow. If vasopressors pass through the topical intradermal tissue at a high level of concentration, the medication can be properly diluted in a large vein. Larger veins provide adequate dilution of the vasopressor and are less likely to spasm. Although the absolute rate of peripheral vasopressor-induced necrosis is unknown, the original rates of ulceration from the peripheral administration of norepinephrine were as high as 46–60%.[19] Similarly, infiltration rates as high as 68% have been reported with the peripheral administration of dopamine in adults.

Vasopressors: **Disperse and Dilute**

First, apply phentolamine 5–10 mg. (except vasopressin and methylene blue)

While applying warm pack, elevate the limb with the extravasation site.

As an alternative, apply topical nitroglycerin 2%. (1^st^ line therapy for vasopressin and methylene blue)

If phentolamine is unavailable, terbutaline 1 mg (phenylephrine is excluded.)

Avoid hyaluronidase monotherapy or cold pack.

***Hyperosmolar Drugs***

There are few, if any, reports in the literature of extravasation injuries due to hypotonic agents; however, hyperosmolar agents pose a major risk for the devolvement of phlebitis and extravasation injuries. Exposure of cells to a hypertonic substance, such as dextrose (10-50%) or radiographic contrast media, causes a direct fluid shift from the intracellular space to the extracellular space. Osmotic stress leads to dysfunction in cellular transport, cell volume dysregulation, direct protein and DNA damage, formation of reactive oxygen species, and the induction of apoptosis.

Regarding potassium or calcium salts, on the other hand, apoptosis occurs due to fluid overload. Also, regarding calcium salts, the tissue can be damaged by the vasoconstriction due to calcium, and calcification can also occur later, mainly in deep tissues. In addition, peripheral TPN (total parenteral nutrition) can cause direct tissue damage when leaking due to being hyperosmotic. Initial presentation of TPN extravasation can include pain, inflammation, and erythema. Extravasation severity can range from benign lipid masses to florid skin necrosis. Therefore, one must be careful not to exceed 900 mOsm/L when administering TPN into peripheral veins. For general extravasation treatments, elevate the affected limb and maintain the elevation while applying compression, and if necessary, administer drug-specific antidotes.

***Acidic and Alkaline Drugs***

Exposure of tissue to an alkaline solution leads to the resultant formation of dissociated hydroxide ions that penetrate tissues extensively. This leads to protein dissolution, collagen destruction, vasoconstriction, fatty acid saponification, cell membrane compromise, and cellular apoptosis.

Acid exposure commonly leads to cellular desiccation, coagulative necrosis, and eschar formation. Edema, vasoconstriction, sloughing, and ulceration are common manifestations of acid-induced tissue injury; however, compared with alkaline exposures, acid-induced injuries are typically less penetrating due to coagulation. Of the two, alkaline exposure has the highest propensity for severe tissue damage due to the deeper tissue penetration.

When the acidic or alkaline infiltrations occur, it is better to conduct adjuvant therapies, like warm compresses. Neutralization should largely be avoided because this can produce exothermic or gas-forming reactions that may worsen tissue injury.  As general treatments for extravasation caused by acidic and alkaline agents, elevate and maintain elevation of the affected limb while applying compresses, and if there is no response to the compression, apply hyaluronidase.

3. Antidotes

If extravasation is still suspected while administering drugs, despite using the methods suggested in Part I, the infusion of drug should be stopped immediately, and reabsorb infiltrated drug as much as possible and then administer drug-specific antidotes. If there is no known antidote or if the amount of the leaked medicinal fluid is small, warm and cold therapy can be applied depending on the properties of the drugs. At this time, the treatments can be classified in two ways according to the properties of the medicinal fluid:

**Localize and Neutralize**: a method to apply antidote by applying cold pack in order to constrict the blood vessels to stop the medicinal fluids from spreading but to make the fluids stay around the affected area; It is applicable primarily to drugs with strong vesicant properties.

**Disperse and Dilute**: a method to lower the concentration level around the affected area by applying warm pack to make the leaked medicinal fluids spread quickly and be absorbed into the surrounding tissue; It is applicable primarily to extravasation due to a vasoconstrictor or hyperosmolar drugs.

***DMSO (Dimethyl Sulfoxide) 50–99%***

DMSO is a topical anti-inflammatory drug that prevents tissue necrosis. In addition, DMSO has a local anesthetic effect, weak bacteriostatic effect, collagen-dissolving action, etc. DMSO is mainly used as an anthracycline antidote and must be applied on skin without blisters immediately after the occurrence of extravasation or as soon as possible.

How to apply:

Mark the extravasation site with a permanent marker.

Apply DMSO with a cotton swab.

Apply every 2 hours during the first 24 hours and for the following 5–7 days at 4 hour intervals.

Apply every 4–6 hours initially and gradually increase the interval (8 hours) to apply twice a day for 7–14 days.

Do not apply occlusive dressing. The area can be covered once it dries.

In order to alleviate topical inflammation, hydrocortisone 1% cream can be applied in between applications of DMSO.

Apply cold pack at the same time.

Precautions: Do not apply to normal skin and wear gloves when applying.

**Do NOT apply to skin with blisters** and avoid strong exposure to sunlight.

Relevant drugs: anthracyclines, dactinomycin, amsacrine, carmustine, dacarbazine, mitomycin C, mitoxantrone, streptozocin.

Side effects: The most common side effects are rash, erythema, dermatitis, pruritus, and sometimes blisters or pain.

***Dexrazoxane***

^As a cyclic derivative of ethylenediaminetetraacetic acid (EDTA), dexrazoxane opens mainly within the cell, shows the actions of chelating formulation, inhibits generation of free radicals, and prevents the cardiotoxicity caused by anthracyclines. In addition, although the exact mechanism is unknown, it is known to reduce cell damage caused by extravasation of anthracycline drugs by inhibiting topoisomerase II. Dexrazoxane has been used to protect the heart against the cardiotoxic side effects of anthracyclines in Korea, but has also approved by the U.S. FDA and the UK as a remedy for extravasation.^

Relevant drugs: anthracyclines (daunorubicin, doxorubicin, epirubicin, idarubicin)

How to apply in accordance with the amount of anthracyclines extravasated:

0–1.5 mL: Do not apply an antidote; apply cold fomentations and hydrocortisone.

1.5–3 mL: Apply topical DMSO, hydrocortisone and cold pack.

> 3 mL: Inject dexrazoxane while applying cold pack. (See Precautions)

How to administer:

Begin within 6 hours from the moment extravasation occurred.

Add a 500 mg vial of the lyophilized powder to 25 mL of sterile water and shake gently to dissolve.

Since the pH of the reconstituted agent is 1.6, dilute it with 200–250 mL of Hartman solution (Phosphate buffered saline).

Administer once a day for 3 days.

Day 1: Continuous intravenous infusion of 1000 mg/m^2^ as soon as possible for 1–2 hours

Day 2: Equal infusion of 1000 mg/m^2^ within three hours after 24 hours from the initial infusion

Day 3: Equal infusion of 500 mg/m^2^ within 3 hours after 24 hours from the second infusion

Daily maximum capacity is 2000 - 2000 - 1000 mg/m^2^.

Precautions:

**Do NOT use dexrazoxane with DMSO.**

Stop applying cold pack 15 minutes prior to the administration of dexrazoxane, and do not apply cold pack for 4 hours after the infusion.

Dexrazoxane is not recommended for patients with liver dysfunction or renal dysfunction.

Reduce the amount by half when creatinine clearance is 40 mL/min or less.

Do not use with live attenuated vaccine.

Do not use with phenytoin.

Serum levels of potassium and sodium must be monitored.

Cytopenia (nadir 11–12 days) may occur because this drug is toxic.

Nausea and vomiting may occur, and liver enzyme levels may increase.

***Hyaluronidase***

As an enzyme that hydrolyzes hyaluronic acid, hyaluronidase increases the distribution of drugs to the connective tissues and promotes the absorption of leaked drugs, and is used to increase a drug’s penetration during subcutaneous injections or intramuscular injections and to promote the reabsorption of body fluids and blood that exist excessively within the tissue.

Relevant drugs: vinca alkaloids, cisplatin, carboplatin, docetaxel, paclitaxel, some non-cytotoxic drugs, monoclonal antibodies, etc.

Reconstitution:

Most of the products that are produced domestically are 1500 IU vials of lyophilized powder or 1500 IU vials of liquid injection formulation and must be diluted to a concentration of 150 units/mL inside a syringe for application on the extravasation site.

Dissolve a 1500 IU vial of hyaluronidase powder in a 1 mL solution of SW (or NS). Use 0.1 mL (=150 units) of SW and 0.9 mL of NS to dilute the hyaluronidase to 150 units/mL.

Or, through the needle left in the extravasation site, inject 1–6 mL of hyaluronidase 1 mL for every 1 mL of the normally leaked medicinal fluids at a concentration of 150 units/mL.

If the amount of drug extravasated is small, take 0.1 mL from 150 units/mL of medicinal fluids, apply with 0.9 mL of NS and dilute to a concentration of 15 units/mL.

For pediatric patients, the standard concentration applied is 15 units/mL.

How to apply:

Divide 150 units (=1 ml) five times (0.2 ml each) and inject subcutaneously in a clockwise direction around the edges of the extravasation site as shown in the figure. Cover the wounded area with dry gauze.

Gently massage the affected area to promote diffusion.

If possible, injection must be done within 60 minutes after the moment extravasation occurred. Use hot fomentations for 1–2 days to help with the quick absorption of hyaluronidase and the diffusion of medicinal fluids.

Apply hydrocortisone cream 1% in the case of inflammation.

Fig 4. Perilesional injections of extravasation site (Onkologie 2013;36:127-135)

***Hydrocortisone***

In the past, SQ and topical application were used to prevent skin necrosis around extravasation sites, using anti-inflammatory action. However, SQ and ID application are not recommended nowadays because a retrospective study reported that fewer surgical interventions were required with a group that did not use corticoid (Schulmeister, 2011a; Langer et al. 2009).

How to apply: In the case of inflammation around the extravasation site, apply hydrocortisone cream 1% twice a day until the erythema disappears. Stop application in the case of blisters.

***Sodium thiosulfate***

Sodium thiosulfate is an antidote that supplies sulfane sulfur molecules to rhodanese inside mitochondria to promote the generation of non-toxic thiocyanate and helps the regeneration of rhodanese to treat cyanide intoxication. In particular, sodium thiosulfate also prevents nephrotoxicity by combining with alkylating antineoplastic agents through nucleophilic reaction to form a non-toxic combination and being excreted in the urine.

Relevant drugs: cisplatin, mechlorethamine

Reconstitution:

Take 1.6 mL of the medicinal fluids 25% and mix with 8.4 mL of SW 8.4 to reconstitute at a concentration of 1/6 M.

Or take 4 mL of the medicinal fluids 10% and mix with 6 mL of SW to reconstitute at a concentration of 1/6 M.

How to apply: Cisplatin

Inject through the IV cannula 2 ml of the reconstituted fluid per cisplatin 100 mg.

Remove the needle.

Inject 0.1 mL of the reconstituted fluid up to 1 mL clockwise along the periphery of the extravasation site.

How to apply: Mechlorethamine

Inject through the IV cannula 2 ml of the medicinal fluid 10% per mg of mechlorethamine.

Remove the needle.

Inject 1/6 M concentration fluid up to 1 mL clockwise along the periphery of the extravasation site.

***Phentolamine***

Phentolamine is an α-adrenergic receptor’s competitive antagonist and prevents skin necrosis and sloughing after venous or peri-venous leakage due to symptomatic drugs, through the mechanism in which the peripheral blood vessel is dilated by relaxing the vascular smooth muscle.

Relevant drugs: epinephrine, norepinephrine, dopamine, dobutamine, phenylephrine

How to apply:

Dilute 5–10 mg in 10 mL of NS 10 and locally inject on the extravasation site within 12 hours.

Inject 0.1 mg/kg for pediatric patients. (Newborns: up to 2.5 mg; adults: up to 10 mg)

Using 25G hypodermic needle, inject along the edge of the affected area several times.

Apply hot fomentations at the same time.

Preventive measures: in order to prevent necrosis caused by drugs, inject phentolamine 10 mg per 1 L of fluid mixed with norepinephrine.

Precautions:

Low blood pressure can be minimized by frequently injecting a small amount for 1–2 hours or longer.

Carefully observe patient for low blood pressure or tachycardia.

Apply as soon as possible within 12 hours from the moment extravasation occurred.

Effect may be insufficient if applied 24 hours or more from the moment extravasation occurred.

***Terbutaline***

As a β2-adrenergic agonist, terbutaline is a drug used to dilate bronchial smooth muscle, but there was a case when it was used as an antidote for extravasation caused by a vasoconstrictor (Am J Emerg Med 1999;17:91-94).

Relevant drugs: norepinephrine, epinephrine, dopamine, dobutamine

How to apply: Dilute 1 mg of terbutaline in 10 mL of NS and locally inject on the extravasation site.

***Topical Nitroglycerin 2% ointment***

Nitroglycerin induces an increase in blood flow by dilating arteries and veins with an action to relax the smooth muscles and improves focal ischemia or skin necrosis caused by vasoconstrictive drugs. Currently, there is no 2% dosage form in Korea; only a 10% ointment formulation is produced for the treatment and mitigation of anal fissure.

Relevant drugs: all vasoconstrictors, such as norepinephrine, epinephrine, dopamine, and dobutamine


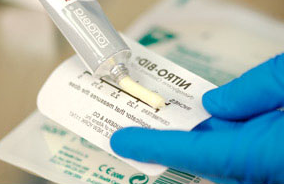
How to apply: apply a 1-inch strip on the area of ischemia as shown in the figure.


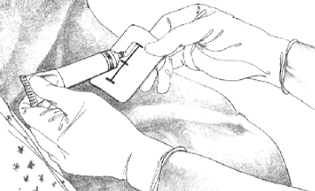


Fig 5. Special pad with measuring strip marked in inches.

Precautions: apply within 12 hours from the moment extravasation occurred and closely monitor for hemodynamic changes when starting.
