# Supplementary material for: Guidelines for the management of extravasation
Source: J Educ Eval Health Prof. 2020 Aug 10;17:21. doi: 10.3352/jeehp.2020.17.21 (PMC7431942; doi:10.3352/jeehp.2020.17.21)
Supplement: Supplementary file 2 — Supplement 2. Special drug management for care of the extravasation. [file jeehp-17-21-suppl2.docx]

**Supplement 2. Special drug management**

In this supplement, the following contents are described in an alphabetical order, regarding proper injection methods and storage of drugs that cause extravasation, proper post-extravasation treatment methods, antidotes, and compressions.

Administration: A process until intravenous injection; how to reconstitute, dilution, iv routes, administration rate and precautions are presented.

SW = Sterile water for injection,

NS = Normal saline for injection,

DW = Dextrose water

SQ = Subcutaneous injection,

IV = Intravenous injection,

IM = Intramuscular injection,

ID = Intradermal injection

Storage: Storage of finished products, stability of reconstituted fluids, and stability of diluted fluids are described.

Treatment methods: Treatment methods are based on Part I [Nursing Intervention Guidelines], and only drug-specific features are briefly described.

Fomentations: Refer to [Fomentations] from Part I [Nursing Intervention Guidelines].

Antidote: Antidotes are indicated if drug-specific antidotes exist.

**Acyclovir**

| Classification | Non-cytotoxic; Irritant or Vesicant; Alkaline agent (pH 11) |
| --- | --- |
| Administration | Dissolve 500 mg vial in 10 mL of SW.  Do not use solvents containing benzyl alcohol.  Dilute in 5DW/NS (concentration of dilution ≤7 mg/mL) and intravenously inject (drip) slowly for one hour.  Ensure that patient is sufficiently hydrated. Risk of phlebitis increases when the concentration of dilution exceeds 10 mg/mL.  Do not use IM and SQ.  Monitor closely for occurrence of phlebitis, and inject alternating injection sites. |
| Storage | Keep at room temperature (15–25℃).  Stabilize the reconstituted fluid at room temperature for 12 hours.  Do not keep the reconstituted fluid or diluent refrigerated due to the possibility of precipitation. |
| Treatment | Apply cold pack first, elevate the limb, and monitor closely.  If not responding to fomentations, the antidote (hyaluronidase) can be tried.  Do not neutralize the extravasation site with acid. |
| Compression | Cold pack |

**Aminophylline**

| Classification | Non-cytotoxic; Vesicant; Alkaline agents (pH 8–10) |
| --- | --- |
| Administration | Dilute in 100 mL of 5DW/NS  Intravenously inject (normal or drip) slowly for 5–10 minutes. |
| Storage | Keep at room temperature. Protect from light. Do not freeze. |
| Treatment | Apply warm pack, elevate the limb, and try the antidote. |
| Antidote | Hyaluronidase |
| Compression | Warm pack |

**Amiodarone**

| Classification | Non-cytotoxic; Vesicant; Acidic agents (pH 3.5–4.5) |
| --- | --- |
| Administration | Dilute 150 mg/amp in 250 mL of 5DW (dilution concentration ≥600 mg/L) and intravenously inject (drip) slowly over 20 minutes to 2 hours.  Be sure to dilute in 5DW only and, if possible, inject through the central venous catheter. |
| Treatment | Apply cold pack first, elevate the limb, and monitor closely.  If not responding to compressions, the antidote (hyaluronidase) can be tried.  Do not neutralize the extravasation site with alkalis.  There is a case where hydrocortisone 1% cream was applied. |
| Compression | Cold pack |

**Amphotericin B (Fungizone®)**

| Classification | Non-cytotoxic; Vesicant (or Irritant); Acidic agents (pH 5–6) |
| --- | --- |
| Administration | Dissolve (5 mg/mL) with 10 mL of SW and dry powder and shake until transparent.  Dilute in 250–500 mL of 5DW and intravenously inject for 4–6 hours. (NS is prohibited.)  Maintain under 0.1 mg/mL when injecting through the peripheral intravenous catheter and under 0.25 mg/mL when injecting through the central venous catheter.  Protection from light while injecting does not affect titers. |
| Storage | Refrigerate. Protect from light.  Protect the reconstituted fluids from light and stabilize for 24 hours at room temperature or for seven days refrigerated.  Protect the diluent from light and stabilize for 24 hours at room temperature or for two days refrigerated. |
| Treatment | Apply cold pack first, elevate the limb, and monitor closely. |
| Compression | Cold pack |

**Arsenic trioxide**

| Classification | Cytotoxic; Non-vesicant |
| --- | --- |
| Administration | Mix with 100–250 mL of 5DW/NS and intravenously inject (continuously) for 1–2 hours.  In the case of symptoms of acute vasoconstriction (low blood pressure, dizziness, grogginess, flushing, headache, etc.), inject slowly by extending the injection time up to 4 hours.  Do not inject via central venous catheter. |
| Storage | Keep at 15–30 ℃.  Stabilize the diluent within 24 hours at room temperature or within 48 hours refrigerated. |
| Treatment | Apply cold pack first, elevate the limb, and monitor closely. |
| Compression | Cold pack |

**Asparaginase**

| Classification | Cytotoxic; Non-vesicant |
| --- | --- |
| Administration | Dissolve in 1–5 mL of SW or NS, shaking slowly.  Use 5-micron filter in order to remove fiber-like particles. (Do not use 0.2-micron filter.)  Dilute in 50–250 mL of NS or 5DW and intravenously inject (drip) for at least 30–60 minutes. |
| Storage | Refrigerate (2 ℃ to 8 ℃).  Use the reconstituted fluid and diluent within 8 hours. |
| Treatment | Immediately stop injection and reabsorb (backflow).  Do not put pressure on the area.  Apply warm pack, elevate the limb, and observe carefully. |
| Antidote | None |
| Compression | Warm pack |

**Bleomycin**

| Classification | Cytotoxic; Non-vesicant |
| --- | --- |
| Administration | IM, SC: Dissolve 15 units in 1–5 mL of SW or NS.  IV: Dissolve 15 units in 5 mL of NS, dilute in 50–1000 mL of NS and intravenously inject slowly. |
| Storage | Keep at room temperature (1 ℃ to 30 ℃).  Stabilize the reconstituted fluid at room temperature or refrigerated for 28 days.  Stabilize the diluent at room temperature for 96 hours or 14 days refrigerated. |
| Treatment | None |
| Compression | None |

**Bendamustine**

| Classification | Cytotoxic; Vesicant (Irritant) |
| --- | --- |
| Administration | Dissolve 25 mg in 10 mL of SW and 100mg in 40 mL of SW, slowly shaking.  Once the medicinal fluid becomes transparent after 5–10 minutes, dilute it in NS 500ml and intravenously inject (drip) for 30–60 minutes while maintaining the final concentration at 0.2–0.6 mg/mL. (NS only) |
| Storage | Keep under 25℃ and protect from light. Dilute immediately after dissolving.  Stabilize the reconstituted fluid for 3.5 hours at 25℃ and the diluent for 2 days refrigerated. |
| Treatment | If the extravasation is confirmed, immediately stop the injection, reabsorb (backflow) briefly and remove the needle.  Apply cold pack and elevate the limb with the extravasation site.  There is no known benefit of the injection of corticosteroid. |
| Antidote | Sodium thiosulfate |
| Compression | Cold pack |

**Bortezomib**

| Classification | Cytotoxic; Non-vesicant |
| --- | --- |
| Administration | SC: Dissolve in 1.4 mL of NS and maintain final concentration at 2.5 mg/mL.  IV: Dissolve in 3.5 mL of NS, maintain final concentration at 1 mg/mL and inject IV bolus for 3–5 seconds. |
| Storage | Keep at 15 ℃ to 30 ℃ and protect from light.  Stabilize the reconstituted fluid at room temperature for up to 8 hours. |
| Treatment | If the extravasation is confirmed, immediately stop the injection, reabsorb (backflow) briefly and remove the needle.  Apply cold pack and topical hydrocortisone 1% during the first 24 hours.  If local reactions remain after 24 hours, apply warm pack and hyaluronidase. |
| Compression | Cold pack |

**Calcium chloride 10%, Calcium gluconate**

| Classification | Non-cytotoxic; Vesicant; Hyperosmolar agents |
| --- | --- |
| Administration | Yeomcaru inj.®: Intravenously injected at a rate under 1 mL/min. (IM and SQ are prohibited due to their potential to cause necrosis.)  Calcium gluconate®: Diluted in a suitable amount of 5DW, NS and injected intravenously. (Do not use IM.)  Precipitated and generated by ethanol; do not use syringes sterilized with ethanol.  Intravenously inject at a rate under 1.5–3.3 mL/min. |
| Storage | Keep at room temperature. Do NOT refrigerate. |
| Treatment | **Early** onset: SQ Hyaluronidase 15 units/mL five times (0.2 mL each with 25GN) along the edge of the extravasation site.  **Delayed** onset (calcinosis cutis): Monitor closely because most calcification naturally disappears. In severe cases, intravenously inject sodium 12.5 g of thiosulfate for 30 minutes and gradually increase up to 25 g by injecting three times a week. |
| Antidote | Hyaluronidase (early), Sodium thiosulfate (delayed) |
| Compression | Warm pack |

**Carboplatin**

| Classification | Cytotoxic; Irritant |
| --- | --- |
| Administration | Dilute in NS or 5DW to final concentration of 0.5–2 mg/mL and IV for 15–60 minutes.  Do not use administering devices that contain aluminum. |
| Storage | 15 ℃ to 30 ℃ (also indicated as under 25 ℃). Protect from light.  After dilution, stabilize at room temperature for up to 8 hours. |
| Treatment | Apply cold pack within 30 minutes from the moment extravasation occurred and continue for 24 hours.  If treatment is administered within 24 hours then a warm pack and consider hyaluronidase would be the treatment of choice.  However if the injury is not treated within 24 hours a cold pack and hydrocortisone cream would then be the appropriate management.^4^  In the case of inflammation, apply hydrocortisone 1% cream every 6 hours for seven days. |
| Antidote | Hyaluronidase, Hydrocortisone |
| Compression | Once the initial inflammation reactions disappear with cold pack, warm pack can be applied in order to diffuse the remaining fluid.  If cold pack were not applied immediately, start applying warm pack within 24 hours. |

**Carmustine**

| Classification | Cytotoxic; Irritant |  |  |
| --- | --- | --- | --- |
| Administration | After dissolving in 3 mL of anhydrous alcohol, apply 27 mL of SW and dissolve to a final concentration of 3.3 mg/mL.  Dilute in 5DW and inject slowly through the central venous catheter for more than 1–2 hours. When injecting high volume, inject for more than 2 hours at the rate of ≤3 mg/m^2^/min or inject twice at an interval of 12 hours.  Because it is adsorbed onto PVC, it is recommended to use non-PVC set, glass, or polyolefin bags. | |  |
| Storage | Keep at 2 ℃ to 8 ℃ and protect from light. Stabilize for seven days refrigerated or at room temperature.  Stabilize the reconstituted fluid for 24 hours refrigerated and the diluent for 8 hours at room temperature if kept from light. | | |
| Treatment | Apply cold pack 4 times a day for 24 hours from the moment extravasation occurred.  If the extravasated amount is < 5 mL, apply topical hydrocortisone 1% cream every 6 hours for seven days. | | |
| Antidote | Hydrocortisone | | |
| Compression | Cold pack | | |

**Cisplatin**

| Classification | Cytotoxic; Irritant (>0.4 mg/mL) |
| --- | --- |
| Administration | For the prevention of nephrotoxicity, inject 1–2 L of the fluid (NS+KCl+MgSO4) before/after the injection, maintain the 24-hour urinary volume at 100 mL/hr or more, dilute the medicinal fluid in NS and intravenously inject for 15–120 minutes.  Use NS only, because 5DW, lactic acid-containing fluid, and amino acid solution cause dissolution.  Precipitated by reacting with aluminum; avoid aluminum-containing devices.  The activity is low in fluids with low chloride ion concentration; use NaCl of 0.3% or higher. |
| Storage | Keep at 15 ℃ to 25 ℃ and protect from light. Beware of crystallization when refrigerating.  Stabilize the diluent at 4 ℃ to 25 ℃ for 72 hours. |
| Treatment | Inject in the IV line 2 mL of **Sodium thiosulfate** 1/6M solution for every 100 mg of the extravasated drug; afterwards, inject 1 mL with SC around the extravasation site (0.1 mL each) and repeat this several times up to 3–4 hours after the onset.  Apply **DMSO** twice a day (8-hour interval) for seven days. Start within at least 10 minutes and do not cover the site. |
| Antidote | Sodium thiosulfate |
| Compression | Once the initial inflammation reactions disappear with cold pack.  If cold pack were not applied immediately, start applying warm pack within 24 hours.  If treatment is administered within 24 hours then a warm pack and consider hyaluronidase would be the treatment of choice.  However if the injury is not treated within 24 hours a cold pack and hydrocortisone cream would then be the appropriate management.^4^ |

**Cladribine**

| Classification | Cytotoxic; Non-vesicant |
| --- | --- |
| Administration | After diluting the medicinal fluid in 500 mL of NS through 0.22-micron filter, inject (drip) for 1–2 hours or inject (continuously) for 24 hours.  Use NS only because the breakdown of main components increases in 5DW. |
| Storage | Keep at 2 ℃ to 8 ℃ and protect from light.  Stabilize the diluent for 24 hours under indoor fluorescent light. |
| Treatment | Apply warm pack 4 times a day for 24 hours from the moment extravasation occurred.  If not responding to compressions, apply hyaluronidase for rapid diffusion. |
| Antidote | Hyaluronidase |
| Compression | Warm pack or None |

**Cyclophosphamide**

| Classification | Cytotoxic; Non-vesicant |
| --- | --- |
| Administration | If you have isotonic agents, dissolve in SW; if not, dissolve in NS to final concentration of 20 mg/mL and inject directly (intravenous, intramuscular, intraperitoneal, intrapleural).  Dilute in NS/5DW and inject intravenously (drip). |
| Storage | Keep at 15 ℃ to 30 ℃. Do not keep Endoxan® injection at temperatures higher than 25 ℃.  Stabilize the reconstituted fluid and diluent for 24 hours at room temperature or for six days refrigerated. |
| Treatment | Apply warm pack 4 times a day for 24 hours from the moment extravasation occurred.  If not responding to compressions, apply hyaluronidase for rapid diffusion. |
| Antidote | Hyaluronidase |
| Compression | Warm pack or None |

**Cytarabine**

| Classification | Cytotoxic; Non-vesicant |
| --- | --- |
| Administration | Dilute in 5DW/NS and IV. |
| Storage | Keep under 25 ℃ and protect from light.  For IV, stabilize the diluent at 25 ℃ for eight days. For IT, immediately use after preparation, considering the biological safety. |
| Treatment | Apply warm pack 4 times a day for 24 hours from the moment extravasation occurred.  If not responding to compressions, apply hyaluronidase for rapid diffusion. |
| Antidote | Hyaluronidase |
| Compression | Warm pack or None |

**Dacarbazine**

| Classification | Cytotoxic; Irritant |
| --- | --- |
| Administration | Dissolve in 9.9 mL of SW to a final concentration of 10 mg/mL, dilute in 250–1000 mL of 5DW/NS and slowly inject intravenously (drip) for longer than 30–60 minutes.  A serious vasostimulant reaction may occur when administering rapidly; when injecting (drip) to prevent angiodynia, protect from light across the drip route. |
| Storage | Keep at 2 ℃ to 8 ℃ and protect from light. Stabilize at room temperature for four weeks.  Stabilize the reconstituted fluid at room temperature for 8 hours (protect from light) or for 72 hours refrigerated.  Use the diluent within 8 hours at room temperature (protected from light) or 24 hours refrigerated. |
| Treatment | Apply cold pack 4 times a day; apply DMSO around the affected area of the skin with no blister as soon as possible.  In order to alleviate the inflammatory reaction, alternate between applications of hydrocortisone 1% cream and DMSO. |
| Antidote | DMSO, hydrocortisone |
| Compression | Cold pack |

**Dactinomycin**

| Classification | Cytotoxic; Vesicant |
| --- | --- |
| Administration | Dissolve in 1.1 mL of SW to a final concentration of 500 mcg/mL and IV push, or  Dilute in 50 mL of 5DW/NS and intravenously inject slowly for 10–15 minutes. Do not use IM or SC. |
| Storage | Keep at 15 ℃ to 30 ℃ and protect from light.  Stabilize the reconstituted fluid for 24 hours at room temperature or refrigerated and the diluent at room temperature within 24 hours. |
| Treatment | Apply cold pack 4 times a day; apply DMSO around the affected area of the skin with no blister, as soon as possible.  In order to alleviate the inflammatory reaction, alternate between applications of hydrocortisone 1% cream and DMSO. |
| Antidote | DMSO, hydrocortisone |
| Compression | Cold pack |

**Daunorubicin**

| Classification | Cytotoxic; Vesicant |
| --- | --- |
| Administration | Dissolve in 10 mL of NS (2 mg/mL) or in 4 mL of SW (5 mg/mL) to  slowly IV push for 1–5 minutes into the NS or 5DW tube injected at a rapid pace, or  dilute in 100 mL of 5DW/NS and intravenously inject (drip) for 15–30 minutes. |
| Storage | Keep at 15 ℃ to 30 ℃ and protect from light.  Stabilize the reconstituted fluid at 15 ℃ to 25 ℃ for four days (protect from light).  Although the diluent is stable for four weeks at 25 ℃ (protected from light), it is recommended to use it within 24 hours. |
| Treatment | Amount extravasated – 0–1.5 mL: apply cold pack and hydrocortisone cream 1%.  Amount extravasated – 1.5–3 mL: apply cold pack, DMSO and hydrocortisone cream 1%.  Amount extravasated – > 3mL: apply cold pack, and inject dexrazoxane. Do NOT use with DMSO. |
| Antidote | Dexrazoxane, DMSO |
| Compression | Cold pack |

**Dextrose 10–50%**

| Classification | Non-cytotoxic; Vesicant; Hyperosmolar agents (504–2520 mOsm/L); |
| --- | --- |
| Administration | 10% must be supplied through peripheral vein, and the higher concentration must be supplied through the central vein because there is irritation upon peripheral administration.  SQ and IM are prohibited for concentrate. |
| Treatment | Apply cold/warm pack, elevate the limb with the extravasation site and try hyaluronidase.  Conduct aseptic surgical flushing with NS on the extravasation site. |
| Antidote | Hyaluronidase |
| Compression | Warm pack, cold pack |

**Diazepam**

| Classification | Non-cytotoxic; Irritant (rarely Vesicant); Hyperosmolar agents (7775 mOsm/L); |
| --- | --- |
| Administration | IV in principle; avoid venulas. Intravenous injection slowly at 5 mg/min. |
| Storage | Keep at room temperature. Protect from light. |
| Treatment | Apply cold pack, elevate the limb with the extravasation site and try the antidote.  Apply hydrocortisone 1% cream in the case of inflammation. |
| Antidote | Hyaluronidase |
| Compression | Cold pack or Warm pack |

**Dobutamine**

| Classification | Non-cytotoxic; Vesicant; Vasopressor |
| --- | --- |
| Administration | Dilute in 250–500 mL of 5DW and inject intravenously. (Central vein is preferred.) |
| Storage | Keep at room temperature. Protect from light. It discolors to pink once oxidized, but there is no difference in efficacy.  Stabilize the reconstituted fluid at room temperature for 48 hours or for seven days refrigerated. |
| Treatment | Apply phentolamine as soon as the extravasation occurs.  Apply warm pack and elevate the limb with the extravasation site.  If there is no phentolamine, use terbutaline instead or try topical nitroglycerin. |
| Antidote | Phentolamine |
| Contraindication | Hyaluronidase monotherapy, ice pack |
| Compression | Warm pack |

**Docetaxel* (One-vial product)**

| Classification | Cytotoxic; Vesicant like Irritant |
| --- | --- |
| Administration | If kept under refrigeration, leave at room temperature for 5 minutes and prepare to 21GN.  Dilute in 250–500 mL of 5DW/NS (final concentration of 0.3–0.74 mg/mL) and inject intravenously (drip) for one hour. Including the injection time, complete the injection within 4 hours and use non-PV bag. |
| Storage | Keep at 2 ℃ to 25 ℃ and protect from light.  Stabilize the diluent at 15 ℃ to 25 ℃ for 4 hours, including the injection time (6 hours for 20 mg/mL products). |
| Treatment | Apply warm pack 4 times a day and apply hydrocortisone 1% cream every 6 hours.  If the amount extravasated is large, apply hyaluronidase. |
| Antidote | Hydrocortisone, hyaluronidase |
| Compression | Warm pack |

* Ways to reconstitute might be different between products. See documentation for applicable product.

**Dopamine**

| Classification | Non-cytotoxic; Vesicant; Vasopressor |
| --- | --- |
| Administration | Dilute in 250–500 mL of 5DW/NS and inject intravenously. (Central vein is preferred.) |
| Storage | Keep at room temperature. Protect from light.  Do not use fluids discolored darker than pale yellow. |
| Treatment | Apply phentolamine as soon as the extravasation occurs.  Apply warm pack and elevate the limb with the extravasation site.  If there is no phentolamine, use terbutaline instead or try topical nitroglycerin. |
| Antidote | Phentolamine |
| Contraindication | Hyaluronidase monotherapy, ice pack |
| Compression | Warm pack |

**Doxorubicin***

| Classification | Cytotoxic; Vesicant |
| --- | --- |
| Administration | Dissolve to 2 mg/mL with SW/5DW and IV push for at least 3–5 minutes or IVPB for 60 minutes.  Or dilute in 50–1000 mL of 5DW, NS and intravenously inject (drip).  5–10 mL flushing before/after the injection in order to prevent extravasation. |
| Storage | (Liquid) Keep at 2 ℃ to 8 ℃ and protect from light; (powder) 15 ℃ to 30 ℃ and protect from light.  Stabilize the reconstituted fluid at 25 ℃ for 24 hours or 48 hours refrigerated.  Stabilize the diluent at room temperature for 40 hours and protect from light. |
| Treatment | Amount extravasated – 0–1.5 mL: apply cold pack and hydrocortisone cream 1%.  Amount extravasated – 1.5–3 mL: apply cold pack, DMSO and hydrocortisone cream 1%.  Amount extravasated – >3mL: apply cold pack and inject dexrazoxane. Do NOT use with DMSO. |
| Antidote | Dexrazoxane, DMSO |
| Compression | Cold pack |

**Epinephrine**

| Classification | Non-cytotoxic; Vesicant; Vasopressor |
| --- | --- |
| Administration | Dilute (4 mcg/mL) 1 mg of the medicinal fluid in 250 mL of 5DW/NS and intravenously inject.  SQ, IM, and IV intracardiac injections are possible. |
| Storage | Keep at room temperature. Protect from light. Sensitive to air; do not use if oxidized and discolored. |
| Treatment | Peripheral intravenous catheter injection:  Apply phentolamine as soon as the extravasation occurs.  Apply hot fomentations and elevate the limb with the extravasation site.  If there is no phentolamine, use terbutaline instead or try topical nitroglycerin.  Autoinjector:  Once extravasation occurs, dilute 0.5–4.5 mg of phentolamine in 5 mL of NS and inject.  If there is no phentolamine, use nitroglycerin 2% or dilute 0.5–1 mg terbutaline in 1 mL of NS and inject. |
| Antidote | Phentolamine |
| Contraindication | Hyaluronidase monotherapy, ice pack |
| Compression | Warm pack |

**Epirubicin**

| Classification | Cytotoxic; Vesicant |
| --- | --- |
| Administration | Dilute in 50–250 mL of 5DW/NS and intravenously inject (drip) for 15–20 minutes, or  slowly IV push for 3–10 minutes. Do not inject within 3 minutes.  In the case of intrabladder injection, dilute in 50 mL of NS and inject (drip) through the catheter. Keep within the bladder for about 1 hour. |
| Storage | Keep at 2 ℃ to 8 ℃ and protect from light.  Stabilize the diluent for 24 hours refrigerated and the diluent for bladder injection at room temperature for 24 hours or 48 hours refrigerated. |
| Treatment | Amount extravasated – 0–1.5 mL: apply cold pack and hydrocortisone cream 1%.  Amount extravasated – 1.5–3 mL: apply cold pack, DMSO, and hydrocortisone cream 1%.  Amount extravasated – >3mL: apply cold pack and inject dexrazoxane. Do NOT use with DMSO. |
| Antidote | Dexrazoxane, DMSO |
| Compression | Cold pack |

**Etomidate (containing propylene glycol 35% v/v)**

| Classification | Non-cytotoxic; Irritant (rarely Vesicant); Hyperosmolar agents |
| --- | --- |
| Administration | Each injection must be done intravenously, slowly for longer than 30 seconds.  Do not give an arterial injection because of the risk of necrosis. |
| Antidote | Hyaluronidase |
| Compression | Cold pack or warm pack |

**Etoposide**

| Classification | Cytotoxic; Irritant |
| --- | --- |
| Administration | Dilute with 250 mL of 5DW/NS or more for every 100 mg of the medicinal fluid and intravenously inject (drip) for 30–60 minutes.  The dilution concentration of 0.4 mg/mL or higher is very unstable, and precipitation can occur within the fluid. In order to dilute a high-volume infusion sufficiently, inject NS/5DW and, at the same time, slowly inject the diluent.  Use non-PVC bag for products containing polysorbate 80. |
| Storage | 1 ℃ to 30 ℃  Stabilize the diluent of 0.4 mg/mL for 24 hours and the diluent 0.2 of mg/mL for 96 hours. |
| Treatment | Treatment is necessary only when a large amount has leaked.  Apply warm pack and elevate the limb with the extravasation site. (US)  In the case of inflammation, apply hydrocortisone 1% cream every 6 hours for seven days. (UK)  If the reaction remains after 24 hours of cold pack, apply warm pack for the following 24–48 hours. (UK) |
| Antidote | Hyaluronidase (US), hydrocortisone (UK) |
| Compression | Warm pack (US), Cold pack (UK) |

**Fludarabine**

| Classification | Cytotoxic; Non-vesicant |
| --- | --- |
| Administration | Inject 2 mL of SW and dissolve by sufficiently shaking for 15 seconds or less. (25 mg/mL)  Dilute in 100–125 mL of 5DW/NS and intravenously inject (drip) for 30 minutes. |
| Storage | Keep at room temperature (1 ℃ to 30 ℃).  The reconstituted fluid must be used within 8 hours because there are no preservatives available.  Stabilize the diluent for 48 hours at room temperature or refrigerated. |
| Treatment | Apply warm pack 4 times a day.  If the amount extravasated is large, apply hyaluronidase. |
| Antidote | Hyaluronidase |
| Compression | Warm pack or None |

**Fluorouracil**

| Classification | Cytotoxic; Irritant |
| --- | --- |
| Administration | Dilute in 50–1000 mL of 5DW/NS and variously IV depending on protocols. |
| Storage | Keep at 1 ℃ to 30 ℃ and protect from light. Crystallization when refrigerated. In the case of crystallization, it can be used by heating at 60 ℃.  Stabilize the diluent at room temperature for 72 hours. |
| Treatment | Apply cold pack 4 times a day for 24 hours and apply hydrocortisone cream 1% every 6 hours.  Once the inflammatory reaction disappears after the initial cold pack, apply warm pack for the following 24–48 hours, and hyaluronidase can be applied in order to quickly diffuse the remaining fluid. |
| Antidote | Hydrocortisone |
| Compression | Cold pack |

**Gemcitabine**

| Classification | Cytotoxic; Non-vesicant |
| --- | --- |
| Administration | Dissolve 200 mg/vial in 5 mL of NS and 1000 mg/vial in 25 mL of NS. (38 mg/mL)  Dilute in 50–500 mL of NS (down to a minimum of 0.1 mg/mL) and intravenously inject (drip) for 30 minutes.  In the case of high volume, it can be injected at the rate of 10 mg/mL/min. |
| Storage | 15 ℃ to 30 ℃  Stabilize the reconstituted fluid at 20 ℃ to 25 ℃ for 24 hours. Keep at room temperature due to the possibility of crystallization when refrigerated.  Stabilize the diluent at 20 ℃ to 25 ℃ for 24 hours. |
| Treatment | Apply warm pack 4 times a day.  If the amount extravasated is large, apply hyaluronidase. |
| Antidote | Hyaluronidase |
| Compression | Warm pack or None |

**Hypertonic sodium chloride (>3%)**

| Classification | Non-cytotoxic; Vesicant; Hyperosmolar agents |
| --- | --- |
| Treatment | Apply cold/warm pack, elevate the limb with the extravasation site, and try hyaluronidase.  Conduct aseptic surgical flushing with NS on the extravasation site. |
| Antidote | Hyaluronidase |
| Compression | Warm pack |

**Idarubicin**

| Classification | Cytotoxic; Vesicant |
| --- | --- |
| Administration | Dissolve in 5 mL of SW (final concentration of 1 mg/mL).  Slowly IV push for 3–5 minutes into the 5DW/NS-injecting tube. (IM and SC are prohibited.)  Dilute the proper amount and inject intravenously (drip) slowly for 10–15 minutes into the 5DW/NS-injecting tube. |
| Storage | Keep at 15 ℃ to 30 ℃ and protect from light.  The reconstituted fluid is stable for 48 hours refrigerated or for 24 hours at 20 ℃ to 25 ℃ but is recommended to be used within 24 hours. |
| Treatment | Amount extravasated – 0–1.5 mL: apply cold pack and hydrocortisone cream 1%.  Amount extravasated – 1.5–3 mL: apply cold f pack, DMSO, and hydrocortisone cream 1%.  Amount extravasated – >3mL: apply cold pack and inject dexrazoxane. Do NOT use with DMSO. |
| Antidote | Dexrazoxane, DMSO |
| Compression | Cold pack |

**Ifosfamide**

| Classification | Cytotoxic; Irritant |
| --- | --- |
| Administration | Dissolve 1000 mg by injecting 20 mL of SW and strongly shaking for 1 minute. (Concentration of 50 mg/mL)  Dilute (0.6–20 mg/mL) in 250–500 mL of 5DW/NS and inject intravenously (drip) for 30 minutes to 2 hours. |
| Storage | Keep under 25 ℃.  Stabilize the reconstituted fluid for 21 days when refrigerating.  The diluent is stable at room temperature for 7 days or for 6 weeks refrigerated but is recommended to be used within 24 hours. |
| Treatment | Apply warm pack 4 times a day for 24 hours.  If the amount extravasated is large, apply hyaluronidase. |
| Antidote | Hyaluronidase |
| Compression | Warm pack |

**Irinotecan**

| Classification | Cytotoxic; Irritant |
| --- | --- |
| Administration | Dilute in 250–500 mL of 5DW (final concentration of 0.12–2.8 mg/mL) and inject intravenously (drip) for 90 minutes or longer.  The medicinal fluid is acidic and more stable with 5DW than NS. |
| Storage | Keep at 1 ℃ to 30 ℃ and protect from light.  Stabilize the diluent at room temperature for 6 hours or for 24 hours refrigerated. (NS diluent can be precipitated when refrigerated.) |
| Treatment | Apply cold pack 4 times a day.  In the case of inflammation, apply hydrocortisone 1% cream every 6 hours for 7 days. |
| Antidote | Hydrocortisone |
| Compression | Cold pack |

**Mannitol 20%**

| Classification | Non-cytotoxic; Vesicant; Hyperosmolar agents (1369 mOsm/L) |
| --- | --- |
| Administration | IV only.  When injecting more than 20%, a 5-micron-filter-attached set must be used. |
| Storage | Easily crystallized; dissolve by heating at 50 ℃. |
| Treatment | Hyaluronidase can be applied while applying cold pack 4 times a day.  Apply hydrocortisone 1% cream  in the case of local inflammation. |
| Antidote | Hyaluronidase |
| Compression | Cold pack |

**Melphalan**

| Classification | Cytotoxic; Irritant |
| --- | --- |
| Administration | Dissolve with 10 mL of the attached solvent. (final concentration of 5 mg/mL)  Dilute in 250–500 mL of NS (dilution concentration of 0.1–0.45 mg/mL), avoid direct injection into peripheral vein but intravenously inject (drip) for 15–30 minutes into the NS tube (speedy injection to central vein). |
| Storage | Keep at 1 ℃ to 30 ℃ and protect from light.  The reconstituted fluid must be used within 60 minutes after being dissolved.  Recommended to be used immediately after dilution. Precipitation when refrigerated. |
| Treatment | Apply warm pack 4 times a day.  If the amount extravasated is large, apply hyaluronidase. |
| Antidote | Hyaluronidase |
| Compression | Warm pack |

**Methotrexate**

| Classification | Cytotoxic; Non-vesicant |
| --- | --- |
| Administration | IM, IV, IT, SQ  Inject in various ways depending on protocols. It can be diluted in 5DW/NS.  IT is possible up to 12 mL by applying NS that does not have preservatives and must be injected immediately after preparation. |
| Storage | Keep under 25 ℃. Protect from light.  Stabilize the diluent at room temperature for 24 hours. |
| Treatment | Apply cold pack 4 times a day for 24 hours and apply hydrocortisone 1% cream every 6 hours.  Once the inflammatory reaction disappears after the initial cold pack, apply warm pack for the following 24–48 hours, and hyaluronidase can be applied in order to quickly diffuse the remaining fluid. |
| Antidote | Hydrocortisone |
| Compression | Cold pack |

**Methylene blue**

| Classification | Non-cytotoxic; Vesicant; Vasopressor |
| --- | --- |
| Administration | Slowly IV push for 5–10 minutes. |
| Treatment | Apply nitroglycerin 2% ointment and repeat every 8 hours as needed.  Apply cold pack and elevate the limb with the extravasation site. |
| Antidote | Topical nitroglycerin |
| Contraindication | Hyaluronidase monotherapy, ice pack |
| Compression | Cold pack |

**Mitomycin C**

| Classification | Cytotoxic; Vesicant |
| --- | --- |
| Administration | Dissolve in the proper amount of SW/NS. (Final concentration of 0.5–1 mg/mL). The manufacturer advises to dissolve in 5 mL of SW.  Dilute in the proper amount of NS (dilution concentration of 0.02–0.04 mg/mL) and intravenously inject (drip) or IV push slowly for 15–30 minutes into the NS tube which is currently injecting. |
| Storage | Keep at 1 ℃ to 30 ℃ and protect from light.  The reconstituted fluid is stable at room temperature for seven days but is recommended to be used within 24 hours.  Stabilize the NS diluent at room temperature for 12 hours. |
| Treatment | Apply cold pack 4 times a day.  Apply DMSO around the affected area with no blister as soon as possible.  In order to alleviate the inflammatory reaction, apply alternatively between hydrocortisone 1% cream and DMSO. |
| Antidote | DMSO, hydrocortisone |
| Compression | Cold pack |

**Mitoxantrone**

| Classification | Cytotoxic; Vesicant (or Irritant) |
| --- | --- |
| Administration | IV only.  Dilute in 50 mL or more of 5DW/NS  and intravenously inject slowly for 5–15 minutes.  Do not inject for a short period of time (3–5 minutes). |
| Storage | Keep at 15 ℃ to 30 ℃ and protect from light.  The reconstituted fluid is stable at room temperature for seven days or for 14 days refrigerated but is recommended to be used within 24 hours.  The diluent is stable at room temperature or refrigerated for seven days, but the manufacturer recommends to use it immediately. |
| Treatment | Apply cold pack 4 times a day.  Apply DMSO around the affected area with no blister as soon as possible.  In order to alleviate the inflammatory reaction, alternate between applications of hydrocortisone 1% cream and DMSO. |
| Antidote | DMSO, hydrocortisone |
| Compression | Cold pack |

**Nafcillin**

| Classification | Non-cytotoxic; Vesicant (or Irritant); Hyperosmolar agents (363 mOsm/L) |
| --- | --- |
| Administration | IM, IV  Intravenously inject (drip) for 30–60 minutes. |
| Treatment | When extravasation occurs, stop the injection, reabsorb (backflow) and remove the needle.  Apply cold pack, elevate the limb with the extravasation site and try hyaluronidase.  (Alternate) Conduct aseptic surgical flushing with NS on the extravasation site. |
| Antidote | Hyaluronidase |
| Compression | Cold pack |

**Nitroglycerin**

| Classification | Non-cytotoxic; Vesicant; Hyperosmolar agents |
| --- | --- |
| Administration | Dilute in 250–500 mL of 5DW/NS and inject intravenously.  Because it is adsorbed onto PVC, dilute in the glass container and use a non-PVC set.  Nitrolingual® infus. can be injected diluted or undiluted. |
| Storage | Stabilize the diluent for 24 hours at room temperature or seven days refrigerated. |
| Treatment | Apply cold/warm pack, elevate the limb with the extravasation site and try hyaluronidase.  Conduct aseptic surgical flushing with NS on the extravasation site. |
| Antidote | Hyaluronidase |
| Compression | Cold or Warm pack |

**Norepinephrine**

| Classification | Non-cytotoxic; Vesicant; Vasopressor |
| --- | --- |
| Administration | Intravenously inject after diluting in 5DW/DS. (NS is prohibited.)  In order to avoid extravasation, use a large-sized vein (preferably central vein). |
| Storage | Keep at room temperature.  Protect from light. Easily oxidized; do not use if it turns amber.  Stabilize the diluent at 25 ℃ for 24 hours. |
| Treatment | Stop the injection, apply warm pack, and elevate the limb.  Phentolamine: Dilute 5–10 mg in 10–20 mL of NS and inject in the affected area. Retry if the symptoms remain.  Terbutaline: Dilute 1 mg in 10 mL of NS and inject in the affected area.  Nitroglycerin: Apply a 1-inch strip of 2% ointment to the affected area and repeat every 8 hours as needed. |
| Antidote | Phentolamine |
| Contraindication | Hyaluronidase monotherapy, ice pack |
| Compression | Warm pack |

**Oxaliplatin**

| Classification | Cytotoxic; Irritant |
| --- | --- |
| Administration | Dilute in 250–500 mL of 5DW (dilution concentration of 0.2 mg/mL or higher) and inject intravenously (drip) for 2–6 hours.  Flushing with 5DW before injection.  Do not use administering devices that contain aluminum.  Solution containing NS/chlorine must not be used because oxaliplatin is dissolved in it. |
| Storage | Keep at room temperature (1 ℃ to 30 ℃).  Stabilize the diluent at 20 ℃ to 25 ℃ for 6 hours or for 24 hours refrigerated. |
| Treatment | Immediately stop the injection, do not conduct flushing, reabsorb (backflow), and remove the needle.  Apply warm pack and elevate the limb with the extravasation site.  Apply hydrocortisone 1% cream every 6 hours.  If the amount extravasated is large, hyaluronidase can be applied. |
| Antidote | Hyaluronidase |
| Compression | Warm pack (due to the properties of this drug, peripheral neuropathy can worsen if cold pack are applied.) |

**Paclitaxel* (Taxol®)**

| Classification | Cytotoxic; Vesicant (Irritant) |
| --- | --- |
| Administration | Dilute in 250–1000 mL of 5DW/NS (dilution concentration of 0.3–1.2 mg/mL) and intravenously inject for 1–96 hours. Varies between protocols.  Use non-PVC bag and IV set. |
| Storage | Keep at 15 ℃ to 30 ℃ and protect from light.  Stabilize the diluent at 15 ℃ to 30 ℃ for 27 hours. |
| Treatment | Apply hyaluronidase while applying warm pack 4 times a day.  Hydrocortisone 1% cream can be applied every 6 hours. |
| Antidote | Hyaluronidase |
| Compression | Warm pack |

*See the manufacturer recommendations due to the difference in methods of administration between dosage forms.

**Pemetrexed**

| Classification | Cytotoxic; Non-vesicant |
| --- | --- |
| Administration | Dissolve 500 mg in 20 mL of NS and 100 mg in 4.2 mL of NS. (final concentration of 25 mg/mL)  Dilute in 100 mL of NS and intravenously inject (drip) over 10 minutes or longer. |
| Storage | Keep at room temperature (15 ℃ to 30 ℃).  Stabilize the reconstituted fluid or diluent for 24 hours refrigerated or for 24 hours at 25 ℃. |
| Treatment | Apply warm pack 4 times a day, and if the amount extravasated is large, apply hyaluronidase. |
| Antidote | Hyaluronidase |
| Compression | Warm pack or None |

**Pentamidine**

| Classification | Non-cytotoxic; Irritant (or Vesicant); Acidic agents (pH 6.5); |
| --- | --- |
| Administration | Dissolve in 3–5 mL of SW/5DW. Do not use NS due to the possibility of precipitation.  Dilute the dissolved medicinal fluid in 50–250 mL of 5DW and intravenously inject slowly for 60–120 minutes. |
| Storage | Keep at 20 ℃ to 25 ℃ and protect from light.  Protect the reconstituted fluid from light and stabilize it at room temperature for 48 hours.  Stabilize the diluent at room temperature for 24 hours. Do not refrigerate due to the possibility of crystallization. |
| Treatment | Apply dry-warm pack first, elevate the limb with the extravasation site and monitor closely.  If not responding to compressions, try hyaluronidase.  Do not neutralize the extravasation site with alkalis. |
| Antidote | Hyaluronidase |
| Compression | Warm pack |

**Phenobarbital (containing propylene glycol 0.678 mL)**

| Classification | Non-cytotoxic; Vesicant; Hyperosmolar agents |
| --- | --- |
| Administration | When injecting intravenously, inject slowly at a maximum injection rate of 50 mg/min or lower.  Do not give an arterial injection due to its strong alkalinity. |
| Treatment | Apply cold/warm pack, elevate the limb with the extravasation site and try the antidote.  Conduct aseptic surgical flushing with NS on the extravasation site. |
| Antidote | None |
| Compression | Cold pack (UK) or warm pack |

**Phenylephrine**

| Classification | Non-cytotoxic; Vesicant; Vasopressor |
| --- | --- |
| Administration | For IM, IV, SQ, and intravenous injection, dilute in 5DW/NS and inject through the vena cava if possible. |
| Storage | Stabilize 5DW diluent at room temperature for 48 hours. |
| Treatment | Stop the injection, apply warm pack, and elevate the limb with the extravasation site.  Phentolamine: Dilute 5–10 mg in 10–20 mL of NS and inject in the affected area. Retry if the symptoms remain.  Nitroglycerin: Apply a 1-inch strip of 2% ointment to the affected area and repeat every 8 hours as needed. |
| Antidote | Phentolamine |
| Contraindication | Hyaluronidase monotherapy, ice pack |
| Compression | Warm pack |

**Phenytoin**

| Classification | Non-cytotoxic; Vesicant; Alkaline agents (pH 10–12) |
| --- | --- |
| Administration | Intravenous infusion rate should not exceed 40 mg/min to prevent extravasation.  Reduce the rate down to 20–25 mg/min for patients with risks, such as old age, high volume, repeated injection, etc.  Use, if possible, a 20-gauge intravenous catheter or higher and maintain it in large-sized veins.  Low blood pressure can occur during rapid injection; maintain the infusion rate and monitor closely.  Generally, dilute the required amount in 100 mL of NS. (Dilution concentration of 1–10 mg/mL). 5DW is prohibited.  Do not mix with other drugs due to its strong alkalinity.  In particular, lowered pH can result in crystallization; therefore, care should be taken in mixing.  Due to high possibility of precipitation, use a 0.22- to 5-micron filter when injecting with IVPB. |
| Storage | Keep at room temperature. Protect from light. *Beware of precipitation when refrigerating.  Stabilize the diluent at room temperature for 4 hours. |
| Treatment | Stop the injection immediately, apply dry-warm pack and elevate the limb with the extravasation site.  If not responding to compressions, try the antidote.  Do not neutralize the extravasation site with acid. |
| Antidote | Divide hyaluronidase 15 units five times and ID along the extravasation site. |
| Compression | Warm pack |

※ Purple Glove Syndrome (PGS)

Definition: In the case of extravascular leakage of the strong alkaline phenytoin medicinal fluid, edema and discoloration gradually appear distally, and purple glove syndrome occurs, in which a patient complains of pain. In severe cases, this causes compartment syndrome, may require surgical treatment, and advances to the following stages:

Stage 1: About 2–12 hours after injection, the affected area turns pale blue or purple inclining to red and causes pain.

Stage 2: About 12–16 hours after discoloration, the area of discoloration gradually widens, and edema begins to appear.

Stage 3: Recovery phase in which edema is improved and discoloration disappears gradually from the edge inward.

**Potassium**

| Classification | Non-cytotoxic; Irritant; Hyperosmolar agents (60 mEq/L = 763 mOsm/L) |
| --- | --- |
| Administration | Do not intravenously inject the medicinal fluid that is not diluted.  IV push cannot be done. It must be diluted in infusion solution and slowly injected intravenously.  Maximum dilution concentration: peripheral vein 8 mEq/L, central vein 30 mEq/100 mL. |
| Storage | Keep at room temperature.  Stabilize the diluent at room temperature for 24 hours. |
| Treatment | Apply cold/warm pack, elevate the limb with the extravasation site, and try the antidote.  Conduct aseptic surgical flushing with NS on the extravasation site. |
| Antidote | Hyaluronidase |
| Compression | Cold or Warm pack |

**Promethazine**

| Classification | Non-cytotoxic; Vesicant; Acidic agents (pH 4.0–5.5) |
| --- | --- |
| Administration | IM and IV can be used, but SQ cannot be used. (IV is not a better route due to tissue damage.)  Must be diluted in 5DW/NS (dilution concentration ≤25 mg/mL); intravenously inject (drip) (max 25 mg/min) into the IV line, which is currently injecting, for 10–15 minutes.  Immediately stop if the patient complains of burning or pain during the injection. |
| Storage | Keep at 20 ℃ to 25 ℃ and protect from light. Stabilize the diluent at room temperature for 24 hours. |
| Treatment | Apply dry-heat fomentations first, elevate the limb with the extravasation site, and monitor closely.  If not responding to fomentations, try the antidote.  Do not neutralize the extravasation site with alkalis. |
| Antidote | Hyaluronidase. |
| Compression | Warm pack or Cold pack |

**Radiographic contrast media**

| Classification | Non-cytotoxic; Vesicant; Hyperosmolar agents |
| --- | --- |
| Administration | <50-mL hypoosmotic contrast agent: monitor closely. Medicinal treatment is not necessary.  >50-mL hypoosmotic contrast agent, hyperosmotic contrast agent: apply hyaluronidase.  Apply cold/warm pack and elevate the limb with the extravasation site.  Conduct aseptic surgical flushing with NS on the extravasation site. |
| Antidote | Hyaluronidase |
| Compression | Warm pack: dilates blood vessels to help the contrast agent quickly circulate and disappear.  Cold pack: prevents the progression of inflammation, eases discomfort, and prevents the intracellular influx of the contrast agent.  *Selection of warm or cold is still debated; animal research found that cold packs were the most effective. NHS recommends warm pack. |

**Raltitrexed**

| Classification | Cytotoxic; Non-vesicant |
| --- | --- |
| Administration | Dissolve with 4 mL of SW (final concentration of 0.5 mg/mL).  Dilute in 50–250 mL of 5DW/NS and intravenously inject (drip) for 15 minutes. |
| Storage | Keep at 2 ℃ to 25 ℃ and protect from light.  Stabilize the reconstituted fluid and diluent for 24 hours refrigerated.  The manufacturer recommends it to be used immediately. |
| Treatment | Apply cold pack 4 times a day for 24 hours and apply hydrocortisone 1% cream every 6 hours.  Once the inflammatory reaction disappears after the initial cold pack, apply warm pack for the following 24–48 hours, and hyaluronidase can be applied in order to quickly diffuse the remaining fluid. |
| Antidote | Hydrocortisone |
| Compression | Cold pack |

**Sodium bicarbonate 8.4%**

| Classification | Non-cytotoxic; Vesicant; Hyperosmolar agents (2000 mOsm/L) |
| --- | --- |
| Administration | In order to avoid extravasation, use a central vein if possible.  Dilute the proper amount in 5DW/NS and intravenously inject for 4–8 hours. |
| Storage | Keep at room temperature.  Use the diluent within 24 hours refrigerated. |
| Treatment | Apply warm pack, elevate the limb with the extravasation site, and try the antidote..  Conduct aseptic surgical flushing with NS on the extravasation site. |
| Antidote | Hyaluronidase |
| Compression | Warm pack |

**Streptozocin**

| Classification | Cytotoxic; Irritant (Vesicant-like) |
| --- | --- |
| Administration | Dissolve in 9.5 mL of 5DW/NS (final concentration of 100 mg/mL).  Dilute in 100 mL or more of 5DW/NS and intravenously inject for 15 minutes to 6 hours. |
| Storage | Keep at 2 ℃ to 8 ℃ and protect from light.  The reconstituted fluid is recommended to be used within 12 hours. (No preservatives available) |
| Treatment | Apply cold pack 4 times a day.  Apply DMSO around the affected area with no blister as soon as possible.  In order to alleviate the inflammatory reaction, alternate between applications of hydrocortisone 1% cream and DMSO. |
| Antidote | DMSO, hydrocortisone |
| Compression | Cold pack |

**Temsirolimus**

| Classification | Cytotoxic; Non-vesicant |
| --- | --- |
| Administration | Dissolve with 1.8 mL of the attached solvent (final concentration of 10 mg/mL). Be careful not to shake hard.  Dilute in 250 mL of NS and intravenously inject (drip) for 30–60 minutes.  Use non-PVC set because polysorbate 80 is contained in the solvent. |
| Storage | Keep at 2 ℃ to 8 ℃ and protect from light.  Stabilize the reconstituted fluid under 25 ℃ for 24 hours.  Stabilize the diluent under 25 ℃ for 6 hours (protect from light). |
| Treatment | Elevate the limb with the extravasation site and, if there is a symptom, apply cold pack 4 times a day.  Apply hydrocortisone 1% cream in the case of erythema or inflammation. |
| Antidote | hydrocortisone |
| Compression | Cold pack |

**Teniposide**

| Classification | Cytotoxic; Irritant |
| --- | --- |
| Administration | Be sure to dilute in 5DW/NS (dilution concentration of 0.1, 0.2, 0.4, 1 mg/mL) and intravenously inject (drip) slowly for 30–60 minutes. Be careful not to IV too fast; flush before/after injection with 5DW/NS.  Inject with non-PVC set due to the possibility of precipitation. |
| Storage | Keep at 2 ℃ to 8 ℃ and protect from light.  Stabilize the diluent (0.1, 0.2, 0.4 mg/mL) at room temperature for 24 hours. Stabilize the diluent 1 mg/mL for 4 hours.  The manufacturer recommends to use it immediately due to the possibility of precipitation at any concentration. |
| Treatment | Stop the injection immediately if extravasation occurs and leave cannula/needle as is.  Slowly reabsorb (backflow) the medicinal fluid that was being injected. At this moment, be careful not to do flushing.  Apply cold pack and try the antidote. |
| Antidote | Hyaluronidase |
| Compression | **Cold pack** |

**Thiopental sodium**

| Classification | Non-cytotoxic; Irritant or Vesicant; Alkaline agents (pH 10–11) |
| --- | --- |
| Administration | Dissolve 500 mg/vial in 20 mL of SW, dilute in 5DW/NS and intravenously inject (continuously).  Do not reconstitute under 2% due to the possibility of hemolysis.  Maintain the dilution concentration at 0.2–0.4%. |
| Storage | Keep at room temperature (20 ℃ to 25 ℃).  Stabilize the reconstituted fluid at room temperature for three days or for seven days refrigerated. |
| Treatment | Primarily, apply cold pack, elevate the limb with the extravasation site, and monitor closely.  If not responding to compressions, try hyaluronidase.  Apply hydrocortisone cream 1% every 6 hours in the case of inflammation. |
| Antidote | Hyaluronidase |
| Compression | Cold pack |

**Thiotepa**

| Classification | Cytotoxic; Non-vesicant |
| --- | --- |
| Administration | Dissolve in 1.5 mL of SW (final concentration of 10 mg/mL), dilute the proper amount with NS and intravenously inject at a rapid rate, using a 0.22-micron filter. Varies between protocols.  In the case of intrabladder injection, dilute in 30–60 mL of NS, inject and residualize for 2 hours. In order to maximize the exposure of the medicinal fluid, change the patient’s posture every 15–30 minutes. |
| Storage | Keep at 2 ℃ to 8 ℃ and protect from light.  Stabilize the reconstituted fluid for 28 days refrigerated or for seven days at 25 ℃. The manufacturer recommends it to be used within 8 hours refrigerated.  Stabilize the diluent of 1 mg/mL at room temperature for 24 hours and the diluent of 3 mg/mL at room temperature for 48 hours. |
| Treatment | Apply warm pack 4 times a day and, if the amount extravasated is large, apply hyaluronidase. |
| Antidote | Hyaluronidase |
| Compression | Warm pack |

**Topotecan**

| Classification | Cytotoxic; Irritant |
| --- | --- |
| Administration | After dissolving with 4 mL of SW (final concentration of 1 mg/mL), dilute in 50–100 mL of 5DW/NS (dilution concentration of 25–50 mcg/mL) and IVPB (intravenous piggyback) for 30 minutes or intravenously inject (drip) for 24 hours. |
| Storage | Keep at 1 ℃ to 30 ℃ and protect from light.  Stabilize the reconstituted fluid for 24 hours refrigerated.  Recommended to be used immediately after being dissolved.  Stabilize the diluent at room temperature for 24 hours. |
| Treatment | Elevate the limb with the extravasation site and, if there is a symptom, apply cold pack 4 times a day.  Apply hydrocortisone cream 1% in the case of erythema or inflammation. |
| Antidote | Hydrocortisone |
| Compression | Cold pack |

**Total Parenteral Nutrition (TPN)**

| Classification | Non-cytotoxic; Vesicant; Hyperosmolar agents |
| --- | --- |
| Administration | Apply warm pack, elevate the limb with the extravasation site, and apply hyaluronidase within 6–12 hours.  There is a case where hyaluronidase was successful in treating newborn extravasation.^2)^  If symptoms still remain after treatment, apply nitroglycerin 2% ointment.  Conduct aseptic surgical flushing with NS on the extravasation site. |
| Antidote | Hyaluronidase |
| Compression | Warm pack |

**Trabectedin**

| Classification | Cytotoxic; Vesicant |
| --- | --- |
| Administration | Completely dissolve 1mg/vial by shaking in 20 mL of SW (final concentration of 0.05 mg/mL).  Dilute in 500 mL of 5DW/NS and intravenously inject (drip) through the central vein for 24 hours. |
| Storage | Refrigerate (2 ℃ to 8 ℃).  Stabilize the reconstituted fluid at room temperature for 30 hours. The manufacturer recommends it to be used immediately.  Stabilize the diluent at room temperature for 30 hours, until the completion of the infusion. |
| Treatment | Stop the injection immediately if extravasation occurs and leave cannula/needle as is.  Do not conduct flushing, slowly reabsorb (backflow) the medicinal fluid, and remove the cannula/needle.  Elevate the limb with the extravasation site and apply fomentations.  Apply hydrocortisone 1% cream in the case of inflammation. |
| Antidote | Currently unknown. |
| Compression | Cold pack |

**Vancomycin**

| Classification | Non-cytotoxic; Irritant (or Vesicant); Acidic agents (pH 4.0) |
| --- | --- |
| Administration | Dissolve 1 g/vial in 20 mL of SW, dilute with 100 mL of fluid for every 500 mg of vancomycin and intravenously inject (drip) intermittently for 60 minutes or more (once). |
| Storage | Stabilize for 14 days at room temperature or refrigerated.  Stabilize the diluent for 14 days refrigerated or seven days at room temperature. |
| Treatment | Apply cold pack first, elevate the limb with the extravasation site, and monitor closely.  If not responding to compressions, try the antidote.  Do not neutralize the extravasation site with alkalis.  Apply hydrocortisone 1% cream in the case of inflammation. |
| Antidote | Hyaluronidase |
| Compression | Cold pack |

**Vasopressin**

| Classification | Non-cytotoxic; Vesicant; Vasopressor |
| --- | --- |
| Administration | Slowly IV push for 5–10 minutes. |
| Treatment | (preferred) **Nitroglycerin**: Apply 1-inch strip of 2% ointment on the affected area and repeat every 8 hours if necessary.  Phentolamine: Dilute 5–10 mg in 10–20 mL of NS and inject in the affected area. Retry if the symptoms remain.  Terbutaline: Dilute 1 mg in 10 mL of NS and inject in the affected area.  Conivaptan, which is the vasopressin antagonist, is pH 3.0 and has a high risk of phlebitis, and more research is needed in order to apply.^2)^ |
| Antidote | Topical nitroglycerin, phentolamine |
| Contraindication | Hyaluronidase monotherapy, ice pack |
| Compression | Cold pack |

**Vinblastine**

| Classification | Cytotoxic; Vesicant |
| --- | --- |
| Administration | Dissolve with the attached solvent (benzyl alcohol-containing NS) or 10 mL of NS (final concentration of 1 mg/mL),  Dilute in 50 mL of 5DW/NS and IV push for 2–3 minutes or intravenously inject (drip) for 5–15 minutes.  The manufacturer recommends to inject into the IV tube, which is currently injecting, for 1 minute without diluting.  Injection of intrathecal is prohibited! (FATAL); inject only through IV.  Risk of extravasation increases if injected for a long time, 30 to 60 minutes, or if the amount of the diluted fluid is more than 100 mL. |
| Storage | Keep at 2 ℃ to 8 ℃ and protect from light.  Stabilize the reconstituted fluid for 28 days refrigerated. |
| Treatment | Stop the injection immediately if extravasation occurs and leave cannula/needle as is.  Do not conduct flushing, slowly reabsorb (backflow) the medicinal fluid and apply the antidote.  Remove the needle, apply warm pack, and elevate the limb with the extravasation site. |
| Antidote | Hyaluronidase: if the needle is not removed, inject 1–6 mL (150 unit/mL) into the IV line.  Generally, inject 1 mL of hyaluronidase for every 1 mL of leaked medicinal fluid.  If the needle is removed, SQ the above volume along the extravasation site.  Or SQ 1 mL along extravasation site (150 units/mL), dividing into 0.2-mL units. |
| Compression | Warm pack |

**Vincristine**

| Classification | Cytotoxic; Vesicant |
| --- | --- |
| Administration | Dilute in 5DW/NS 20–50 mL bag and intravenously inject rapidly for 5–10 minutes (preferred), or  IV push for 1–2 minutes or intravenously inject (drip) for 24 hours.  Injection of intrathecal is prohibited! Inject only through IV. |
| Storage | Keep at 2 ℃ to 8 ℃ and protect from light.  The diluent is stable for seven days refrigerated or two days at room temperature but is recommended to be used within 24 hours. |
| Compression | Warm pack |

*See vinblastine for treatment methods and antidote.

**Vinorelbine**

| Classification | Cytotoxic; Vesicant |
| --- | --- |
| Administration | Apply the proper amount of 5DW/NS, dilute 1.5–3 mg/mL (syringe) or 0.5–2 mg/mL (bag) and intravenously inject at a fast rate for 6–10 minutes. (Up to 30 minutes)  Risks of phlebitis and pain increase when injecting for a long time.  Injection of intrathecal is prohibited! Inject only through IV. |
| Storage | Keep at 2 ℃ to 8 ℃ and protect from light. Stabilize for 72 hours at room temperature.  Stabilize the diluent at room temperature for 24 hours. |
| Compression | Warm pack |

*See vinblastine for treatment methods and antidote.
