# Supplementary material for: Guidelines for the management of extravasation
Source: J Educ Eval Health Prof. 2020 Aug 10;17:21. doi: 10.3352/jeehp.2020.17.21 (PMC7431942; doi:10.3352/jeehp.2020.17.21)
Supplement: Supplementary file 5 — Supplement 5. Photographic documentation of extravasation case. [file jeehp-17-21-suppl5.docx]

Supple 5. Photographic documentation of extravasation case

Extravasation

Reporter : ________________

| 1. Date of Request: ______________________ Department: ___________ Physician: ___________ 2. IV device-related information    - 1. IV device insertion date/time: ____/____/____ _ _ _: _ _ _      2. IV device inserted by: ____________      3. IV device type   Peripheral intravenous catheter ( G) Non-tunnel ( ) Tunnel ( ) Implanted port PICC   1. Extravasation drug-related information    - 1. Drug names/dosage: ___________/________      2. Vesicant drug: Yes No      3. Drug administered on: ____/___/____ (administered by:_______________)      4. Chemo_regimen: ___________________      5. Administration path: IV push Piggyback IM Flush only other_____________      6. Blood backflow confirmed during administration: Yes No      7. Patient’s symptoms and expression during administration: ________________________________ 2. Extravasation site assessment   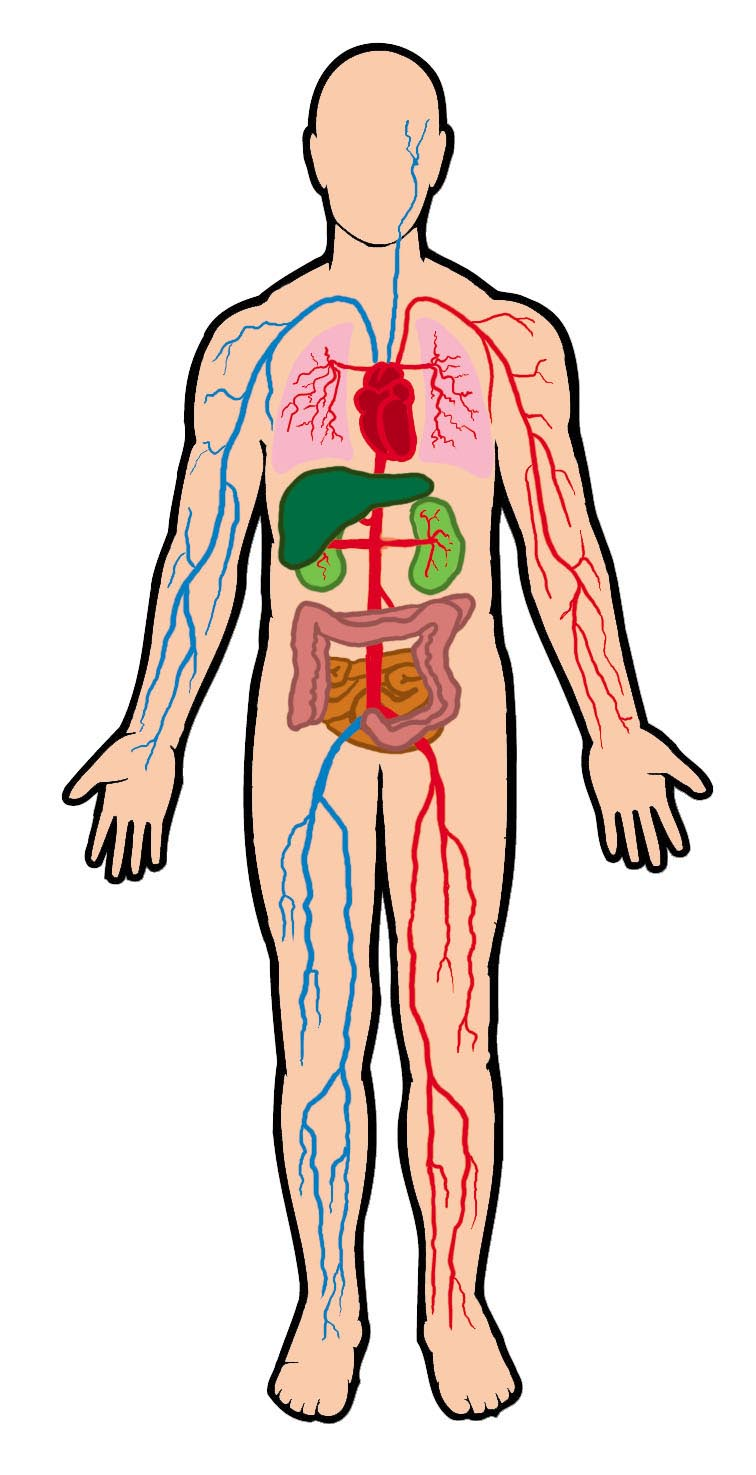   - - 1. Assessed on: _________(year) _____(month) ______(day) (D_____)     2. Extravasation site   Rt Lt  Upper extremity Lower extremity  Internal External  Details: _______________________   - - 1. Description of extravasation site’s condition (skin condition, size, etc.)   < Details >  _____________________________________________   1. Therapeutic interventions    - Administration of antidote: Yes _____________ No    - Intervention contents: _____________________________________________________________    - Medical photography: Yes No    - Results: Resolved after emergency treatment Resolved after single medication Skin graft required F/U Loss |  |
| --- | --- |
| 1. Follow-up  \| Date \| / / \| / / \| / / \| / / \| / / \| / / \| / / \| \| --- \| --- \| --- \| --- \| --- \| --- \| --- \| --- \| \| Extravasation occurred on \| D( ) \| D( ) \| D( ) \| D( ) \| D( ) \| D( ) \| D( ) \| \| Color \|  \|  \|  \|  \|  \|  \|  \| \| Integrity \|  \|  \|  \|  \|  \|  \|  \| \| Skin temp. \|  \|  \|  \|  \|  \|  \|  \| \| Edema \|  \|  \|  \|  \|  \|  \|  \| \| Mobility \|  \|  \|  \|  \|  \|  \|  \| \| Pain \|  \|  \|  \|  \|  \|  \|  \| \| Fever \|  \|  \|  \|  \|  \|  \|  \| \| Comment \|  \|  \|  \|  \|  \|  \|  \|  \| Date \| / / \| / / \| / / \| / / \| / / \| / / \| / / \| \| --- \| --- \| --- \| --- \| --- \| --- \| --- \| --- \| \| Extravasation occurred on \| D( ) \| D( ) \| D( ) \| D( ) \| D( ) \| D( ) \| D( ) \| \| Color \|  \|  \|  \|  \|  \|  \|  \| \| Integrity \|  \|  \|  \|  \|  \|  \|  \| \| Skin temp. \|  \|  \|  \|  \|  \|  \|  \| \| Edema \|  \|  \|  \|  \|  \|  \|  \| \| Mobility \|  \|  \|  \|  \|  \|  \|  \| \| Pain \|  \|  \|  \|  \|  \|  \|  \| \| Fever \|  \|  \|  \|  \|  \|  \|  \| \| Comment \|  \|  \|  \|  \|  \|  \|  \|   **<Extravasation Assessment Tool>**   \| Score  Evaluation items \| 0 \| \| 1 \| 2 \| 3 \| 4 \| \| --- \| --- \| --- \| --- \| --- \| --- \| --- \| \| Color \| Normal \| \| Pink \| Red \| Blanched \| Blackened \| \| Integrity \| Unbroken \| \| Blistered \| Superficial skin loss \| Tissue loss, exposing subcutaneous tissue \| Tissue loss, exposing muscle, /bone with a deep crater or necrosis \| \| Skin temp. \| Normal \| \| Warm \| Hot \|  \|  \| \| Edema \| Absent \| \| Non-pitting \| Pitting \|  \|  \| \| Mobility \| Full \| \| Slightly limited \| Very limited \| Immobile \|  \| \| Pain \| Rate on 0-10 scale \| \| \| \| \| \| \| Fever \| Normal \| Elevated (highest value during 24 hours) \| \| \| \| \| | |
